# Effectiveness and safety of dipeptidyl peptidase 4 inhibitors in the management of type 2 diabetes in older adults: a systematic review and development of recommendations to reduce inappropriate prescribing

**DOI:** 10.1186/s12877-017-0571-8

**Published:** 2017-10-16

**Authors:** Gisela Schott, Yolanda V Martinez, R. Erandie Ediriweera de Silva, Anna Renom-Guiteras, Anna Vögele, David Reeves, Ilkka Kunnamo, Minna Marttila-Vaara, Andreas Sönnichsen

**Affiliations:** 1 Drug Commission of the German Medical Association, Berlin, Germany; 2 NIHR School for Primary Care Research, Manchester Academic Health Science Centre, University of Manchester, Manchester, England; 3 Family Medicine Unit, Faculty of Medicine, University of Colombo, Colombo, Sri Lanka; 4 Institute of General Practice and Family Medicine, Witten/Herdecke University, Witten, Germany; 5 Department of Geriatrics, University Hospital Parc de Salut Mar, Barcelona, Spain; 6 South Tyrolean Academy of General Practice, Bolzano, Italy; 7 Duodecim Medical Publications Ltd, Helsinki, Finland

**Keywords:** Systematic review, Dipeptidyl-peptidase IV inhibitors, Type 2 diabetes mellitus, Inappropriate prescribing

## Abstract

**Background:**

Preventable drug-related hospital admissions can be associated with drugs used in diabetes and the benefits of strict diabetes control may not outweigh the risks, especially in older populations. The aim of this study was to look for evidence on risks and benefits of DPP-4 inhibitors in older adults and to use this evidence to develop recommendations for the electronic decision support tool of the PRIMA-eDS project.

**Methods:**

Systematic review using a staged approach which searches for systematic reviews and meta-analyses first, then individual studies only if prior searches were inconclusive. The target population were older people (≥65 years old) with type 2 diabetes. We included studies reporting on the efficacy and/or safety of DPP-4 inhibitors for the management of type 2 diabetes. Studies were included irrespective of DPP-4 inhibitors prescribed as monotherapy or in combination with any other drug for the treatment of type 2 diabetes. The target intervention was DPP-4 inhibitors compared to placebo, no treatment, other drugs to treat type 2 diabetes or a non-pharmacological intervention.

**Results:**

Thirty studies (reported in 33 publications) were included: 1 meta-analysis, 17 intervention studies and 12 observational studies. Sixteen studies were focused on older adults and 14 studies reported subgroup analyses in participants ≥65, ≥70, or ≥75 years. Comorbidities were reported by 26 studies and frailty or functional status by one study. There were conflicting findings regarding the effectiveness of DPP-4 inhibitors in older adults. In general, DPP-4 inhibitors showed similar or better safety than placebo and other antidiabetic drugs. However, these safety data are mainly based on short-term outcomes like hypoglycaemia in studies with HbA1c control levels recommended for younger people. One recommendation was developed advising clinicians to reconsider the use of DPP-4 inhibitors for the management of type 2 diabetes in older adults with HbA1c <8.5% because of scarce data on clinically relevant benefits of their use. Twenty-two of the included studies were funded by pharmaceutical companies and authored or co-authored by employees of the sponsor.

**Conclusions:**

Other than the surrogate endpoint of improved glycaemic control, data on clinically relevant benefits of DPP-4 inhibitors in the treatment of type 2 diabetes mellitus in older adults is scarce. DPP-4 inhibitors might have a lower risk of hypoglycaemia compared to other antidiabetic drugs but data show conflicting findings for long-term benefits. Further studies are needed that evaluate the risks and benefits of DPP-4 inhibitors for the management of type 2 diabetes mellitus in older adults, using clinically relevant outcomes and including representative samples of older adults with information on their frailty status and comorbidities. Studies are also needed that are independent of pharmaceutical company involvement.

**Electronic supplementary material:**

The online version of this article (doi:10.1186/s12877-017-0571-8) contains supplementary material, which is available to authorized users.

## Background

Diabetes is a prevalent chronic disease worldwide. The International Diabetes Federation estimated the prevalence of diabetes to be 8.8% in adults 20 to 79 years old and close to 20% in people aged over 65 years [1]. Diabetes and its complications are an important cause of morbidity and mortality, and people with diabetes have substantially reduced life expectancy [2]. Duration of diabetes and the degree of metabolic control are important factors determining the prognosis for people with diabetes [3]. However, drugs used in diabetes are one of the most commonly used drug groups associated with preventable hospital admissions related to adverse drug events and overtreatment, especially in older populations [4]. Furthermore, some studies suggest that strict metabolic control may not be advisable for older and frail people, because the benefits may not outweigh the risks of the treatment [5].

Dipeptidyl peptidase-4 (DPP-4) inhibitors are oral agents used for the pharmacological treatment of adults with type 2 diabetes mellitus. The main representatives of this class are sitagliptin, saxagliptin, vildagliptin, linagliptin, teneligliptin and alogliptin.

DPP-4 is a protease involved in glucagon-like peptide-1 (GLP-1) inactivation. By inhibiting the enzyme, DPP-4 inhibitors prolong and enhance the activity of GLP-1 [6]. GLP-1 exerts its main effects by stimulating glucose-dependent insulin release, slowing gastric emptying, reducing food intake, and decreasing postprandial glucagon excretion.

The approved indications for DPP-4 inhibitors are limited to patients for whom diet and exercise do not provide adequate glycaemic control. In addition, first line use of metformin is recommended unless metformin is not tolerated or contraindicated [7], and this is also the case for older populations [8]. In clinical guidelines, DPP-4 inhibitors are recommended only as a second or third line treatment [7, 9, 10].

A systematic review has shown that in patients with type 2 diabetes, who do not achieve the glycaemic targets with metformin alone, DPP-4 inhibitors can lower HbA1c to the same extent as sulfonylureas or pioglitazone, with neutral effect on body weight [11]. However, this systematic review did not report the age ranges of the participants in the included studies. Furthermore, HbA1c and body weight are arguably only surrogate outcomes for more clinically relevant endpoints such as physical and mental status, quality of life, and life expectancy.

Data on long-term risks and benefits of DPP4-inhibitors are scarce. Only three randomised controlled trials of DPP4-inhibitors have looked at clinically relevant endpoints for an observation period of at least 18 months [12–14]. These trials respectively compared saxagliptin, sitagliptin and alogliptin to placebo (alongside existing therapy) [12–14]. However, all these trials report only minimal, or no, results specific to older participants (65 years or more) [12–14]. This reflects the common problem that older people, despite being major users and potentially having a different response to pharmaceutical interventions, are under-represented in most drug trials [15, 16], and that clinical guidelines often base their recommendations on evidence mostly from younger populations [17]. To the best of our knowledge, no systematic review has evaluated the specific evidence on the use of DPP-4 inhibitors in older populations.

The objectives of this systematic review (SR) are therefore:

- to systematically review the literature on the risks and benefits of the use of DPP-4 inhibitors in the treatment of type 2 diabetes in older adults,
- to critically assess the quality of the evidence identified, and
- to develop recommendations in relation to discontinuation or dose-adjustment of DPP-4 inhibitors in the treatment of type 2 diabetes in older adults.

The recommendations developed will be used in an electronic decision support tool in the PRIMA-eDS project [18].

## Methods

This SR was conducted following an adaption of the methods recommended by both the Cochrane Handbook for Systematic Reviews of Interventions [19] and the Preferred Reporting Items for Systematic Reviews and Meta-Analyses (PRISMA) [20].

For undertaking this SR, as one of a planned long-term series of SRs on the efficacy and safety of commonly prescribed drugs in older people, we purposely developed an efficient methodology that does not compromise quality. A full description of our methods has been published [21], but in brief we developed a four-stage approach by which we initially search for systematic reviews and meta-analyses (search 1 and 2) and only if necessary move on to searching for individual studies (search 3A and 3B; see Search method below). Each subsequent stage is only undertaken if the accumulated evidence from the previous stages is deemed not sufficient, or of sufficient quality, to enable evidence based recommendations to be made. A specific protocol for the present SR was prepared and is available from the authors upon request.

### Study inclusion criteria

#### Types of studies

In line with our methodology, in a staged fashion we included systematic reviews, meta-analyses, controlled interventional studies and observational studies reporting on risks and benefits of the use of DPP-4 inhibitors in the treatment of type 2 diabetes in older adults. We excluded conference abstracts, pooled analyses, editorials, opinion papers, case reports, case series, narrative reviews, letters, and qualitative studies.

#### Type of participants

We explicitly searched for studies on older people (≥65 years old) with type 2 diabetes. Our specific age criteria for inclusion varied according to study design:

For systematic reviews and meta-analyses (any of the following criteria):

- Overall mean or median age ≥ 65 years;
- Overall mean or median age < 65 but subgroup analysis reporting on participants ≥65 years;
- Overall mean or median age not reported but 80% or more of the included studies reported a mean or median age ≥ 65 years.

For controlled interventional studies and observational studies (any of the following criteria):

- ≥80% of participants ≥65 years;
- <80% of participants ≥65 years but subgroup analysis reporting on participants ≥65 years.

#### Types of interventions

We included studies reporting on the efficacy and/or safety of any DPP-4 inhibitor for the management of type 2 diabetes. Studies were included irrespective of DPP-4 inhibitors prescribed as monotherapy or in combination with any other drug for the treatment of type 2 diabetes. We included studies comparing DPP-4 inhibitors versus placebo, no treatment, other drugs to treat type 2 diabetes or a non-pharmacological intervention.

#### Types of outcomes

We included studies that used any of the following clinically relevant endpoints as primary or secondary outcomes: hypoglycaemia, adverse events, quality of life, mortality, life expectancy, a related hospitalisation, cognitive impairment or cognitive status, functional impairment or functional status, cardiovascular events including stroke, renal failure, composite end points including any of the above, any of the above evaluated as safety endpoints. Studies reporting other outcomes considered as clinically relevant were also considered for inclusion. We excluded studies evaluating only glycaemic control, changes in HbA1c levels or other endpoints considered to be not clinically relevant. To aid interpretation of findings we have classified outcomes into two tiers according to their anticipated impact on longer-term health and quality of life: Tier 1 outcomes generally have shorter-term impact and include hypoglycaemia and adverse events (including serious adverse events); Tier 2 outcomes have longer-term impact and include, but aren’t limited to, cardiovascular and cerebrovascular events, related hospitalisations, and death.

#### Setting

We included any setting reporting on the management of type 2 diabetes using DPP-4 inhibitors.

#### Language

We did not apply any language restriction to the search but we only included studies that could be read by the research team (languages: English, German, Finish, Italian, and Spanish).

### Search method

Database searches were conducted by YVM. We started searching for systematic reviews and meta-analyses (search 1 and 2). During study selection under search 1 and 2, we identified eligible individual studies from excluded systematic reviews and meta-analyses and transferred these to the Search 3A list for potential inclusion. The list of studies in Search 3A was checked for inclusion following the procedures described below under “Selection of studies”. Only one relevant meta-analysis was found from Searches 1 and 2. However, this meta-analysis covered just one type of DPP-4 inhibitor (linagliptin). Therefore, we conducted Search 3B for individual studies published in the last 10 years (2005–2015) [21]. Detailed information about databases and search dates is summarised below:

- Search 1 was conducted on 03 December 2015 in the Cochrane Database of Systematic Reviews (OVID interface, 2005 to November 2015) and the Database of Abstracts or Reviews of Effects (DARE, OVID interface, 1991 to 2nd Quarter 2015).
- Search 2 was conducted on 03 December 2015 in MEDLINE (OVID interface, 1946 to November Week 3 2015), EMBASE (OVID interface, 1974 to 2015 December 02), Health Technology Assessment (HTA, OVID interface 2001 to 4th Quarter 2015) and International Pharmaceutical Abstracts (IPA, OVID interface 1970 to November 2015).
- Search 3A consisted of controlled intervention and observational studies from systematic reviews and meta-analysis not included in searches 1 and 2 but containing eligible studies.
- Search 3B was conducted on 7 December 2015 in MEDLINE (OVID interface, 2005 to November Week 3 2015), EMBASE (OVID interface, 2005 to 2015 December 04), HTA (OVID interface 2005 to 4th Quarter 2015) and IPA (OVID interface 2005 to November 2015).

In addition to database searches, we checked the references of included reviews and studies following the procedures described later under “Selection of studies”. A list of excluded studies after full-text check with reasons for exclusion is provided in Additional file 1.

The PICOS-framework was used to develop the search terms (population: older people with type 2 diabetes, intervention: DPP-4 inhibitors, comparison: any, outcomes: see list above “Types of outcomes” and study design: systematic reviews, meta-analyses, controlled interventional studies and observational studies). We also created search filters specific to different study designs and each filter is described in detail in the protocol [21]. Additional file 2 shows the full search terms for each search (i.e. Searches 1, 2 and 3B).

### Data management

Literature search results were uploaded to the Endnote X7 reference management software. Endnote was used to import search results and to de-duplicate references.

### Selection of studies

First, two independent reviewers assessed titles and abstracts from each search and identified studies to include. Second, full manuscripts were obtained for all titles and abstracts that met the inclusion criteria or where there was any uncertainty for inclusion. GS, AV, YVM and REED were involved in this task. Reviewers agreed on which articles should be included and ARG acted as arbitrator when GS, AV, YVM and REED could not reach a full consensus.

### Data extraction

GS, YVM and REED independently conducted data extraction from each study using a standardised and piloted data collection form which has been published alongside the protocol [21]. GS, YVM and REED checked each other’s data extraction to look for completeness and accuracy. The data extraction form collected information related to the study design and aim, characteristics of participants (age, sex, setting, comorbidities, use of concomitant medications, functional status, frailty, and cognitive status), the intervention (i.e. DPP-4 inhibitors) and comparison, time to follow-up, and reported outcomes. We also collected information on the involvement of pharmaceutical companies in the included studies.

### Quality appraisal

We used three validated assessment tools to assess the quality of the evidence from each included study: for systematic reviews/meta-analyses the Assessment of Multiple Systematic Reviews tool (AMSTAR) [22, 23], for intervention studies the Cochrane Collaboration’s tool for assessing risk of bias [19], and for observational studies the Critical Appraisal Skills Programme (CASP) [24, 25].

An overall rating for each study was made based on study limitations as suggested by Guyatt et al. (2008) [26], starting with high quality for randomised trials without important limitations (such as lack of allocation concealment; lack of blinding, large loss at follow-up, unmet intention to treat analysis, stopping early for benefit; and failure to report outcomes) and low quality for observational studies without important limitations.

### Dealing with duplicate and companion publications

We included all relevant data from publications relating to a single primary study. Due to our staged approach, it was possible that a publication that was part of an included systematic review or meta-analysis, would also be included as a separate individual study, resulting in a risk of “double-counting”. Any such instances have been identified and reported and taken account of in our synthesis of results.

### Data synthesis

A narrative synthesis describing all included systematic reviews, meta-analyses, intervention and observational studies, participants and findings was carried out. The included studies were highly heterogeneous regarding type of DPP-4 inhibitors, comparison (form of control treatment or placebo), length of follow-up and outcome definition (e.g. types of adverse events included); therefore no additional meta-analyses were performed. The quality of the included studies is also reported.

### Identification of “references of interest” for the development of recommendations

During the search process, GS, and YVM identified and collected additional material relevant to the development of recommendations according to the methodology described by Martinez/Renom - Guiteras et al. (2017) [21].

### Development of recommendations

Included studies and additional references were summarised in a document that was used in team meetings to develop recommendations on when the use of DPP-4 inhibitors could be safely discontinued or the dosage reduced in the management of type 2 diabetes in older people [21]. Each recommendation was given a rating for strength (weak or strong) and quality (low, moderate or high) of evidence following the GRADE methodology [26–28].

## Results

### Results of the search

**Fig. 1:**
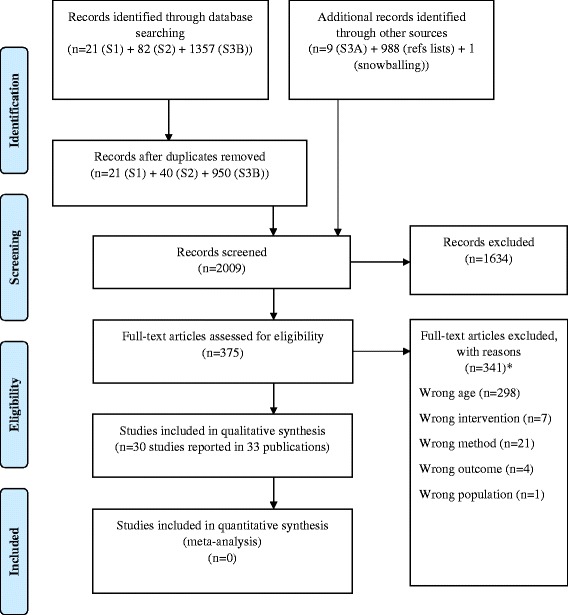
Preferred Reporting Items for Systematic Reviews and Meta-Analyses (PRISMA) flow diagram. *Additional file 1 includes the list of excluded studies with reasons

We identified 1460 records through initial database searching (21 from search 1, 82 from search 2, 9 from search 3A and 1357 from search 3B). Additionally, we identified 988 records from reference lists of included studies, and one further study by snowballing. After removing duplicates, we screened 2009 records and excluded 1634 records after checking titles and abstracts. We assessed 375 full-texts for eligibility and excluded 341 records. We included 30 studies reported in 33 publications. The PRISMA flow diagram is presented in Fig. 1.

### Included studies

Table 1 shows details of included studies. Thirty studies met our inclusion criteria. These studies included more than 273,358 participants ≥65 years. The largest had 141,322 participants and the smallest 60 participants.

**Table 1:** Summary of study characteristics

Authors and publication year (search of identification)	Type of study	Aim	Sample size and information about the amount of older participants*	Follow-up	Outcomes and measurement tools if applicable
Johansen et al. 2012 [58] (Search 3B)	Pre-specified, prospective, and adjudicated meta-analysis of a phase 3 programme	To determine the cardiovascular safety of linagliptin.	RCTs (phase 3): 8 P: 5239 *P* ≥ 65 years: 1478 P using linagliptin: 929 P using comparators: 549	P using linagliptin: 175 (1, 617) ^a^ days P using comparators: 179 (1, 619) ^a^ days for total comparators (169 [1, 367] ^a^days for placebo and 409 [3, 619] ^a^ days for active comparators)	Composite of CV death (including fatal stroke and fatal MI), non-fatal stroke, non-fatal MI, and hospitalisation for UAP. Composites of: (i) CV death, non-fatal stroke, and non-fatal MI; (ii) all adjudicated CV events which included CV death, non-fatal stroke, non-fatal MI, UAP with or without hospitalisation, SAP, and TIA; and (iii) FDA-defined custom MACE derived from 34 unadjudicated MedDRA preferred terms for stroke and MI. Individual adjudicated components (as listed above) and total mortality.
Banerji et al. 2010 [29] (Search 3B)	Retrospective analysis of the GALIANT study which is a multicentre, randomised, open-label study	To assess the safety profile of vildagliptin compared to TZD as an add-on to metformin in patients with T2DM with mild renal impairment and with normal renal function.	P: 2613 *P* ≥ 65 years: 519 P normal renal function: 248 P using vildagliptin 100 mg + metformin: 157 P using TZD + metformin: 91 P mild impaired renal function: 271 P using vildagliptin 100 mg + metformin: 184 P using TZD + metformin: 87	12 weeks	Adverse events
Barnett et al. 2013 [31] (Search 3B)	Randomised, double-blind, placebo-controlled trial	To assess the effectiveness of linagliptin in elderly patients with type 2 diabetes.	P: 241 *P* ≥ 65 years: 241 P using linagliptin: 162 P using placebo: 79	24 weeks	Incidence and intensity of AEs, withdrawals because of AEs, hypoglycaemia, cardiovascular events, and changes in vital signs, laboratory variables, and background treatment.
Barzilai et al. 2011 [30] (Search 3A)	Randomised, double-blind, placebo-controlled trial	To assess efficacy and safety, and tolerability of sitagliptin monotherapy in elderly patients.	P: 206 *P* ≥ 65 years: 206 P using sitagliptin: 102 P using placebo: 104	24 weeks	AEs, SAEs, and hypoglycaemia.
Chien et al. 2011 [32] (Search 3B)	Randomised, open-labelled, parallel-group study	To examine the effectiveness and tolerability of add-on sitagliptin in elderly T2DM patients with inadequate control to existing OAD combination regimen.	P: 97 *P* ≥ 65 years: 97 P using sitagliptin + OAD combinations (sulfonylurea, metformin, and alpha glucosidase inhibitors): 49 P using OAD combinations (sulfonylurea, metformin, and alpha glucosidase inhibitors): 48	24 weeks	AEs.
Ferrannini et al. 2009 [54] (Search 3B)	Multicentre, randomised, double-blind, active-controlled study	To evaluate the positioning of DPP-4 inhibitors as add-on to metformin when metformin alone is not sufficient to achieve glycaemic control, the long-term efficacy and safety of vildagliptin vs. SU was examined.	P: 2789 *P* ≥ 65 years: 712 P using vildagliptin: 351 P using glimepiride: 361	52 weeks	AEs.
Fonseca et al. 2008 [56] (Search 3B)	Multi-centre, double-blind, parallel-group, randomised study	To report of longer term data from a clinical trial, undertaken to assess the efficacy and safety of vildagliptin therapy over one year in patients with long-standing T2DM that was inadequately controlled by insulin therapy.	P: 200 *P* ≥ 65 years: 60 P using vildagliptin 100 mg/d + insulin: 32 P using placebo + insulin / vildagliptin 50 mg/d + insulin: 28	52 weeks	All AEs, SAEs, and hypoglycaemic events.
Green et al. 2015 [13] (Snowballing)	Randomised, double-blind, placebo-controlled study (Trial Evaluating Cardiovascular Outcomes with Sitagliptin [TECOS])	To assess the long-term cardiovascular safety of adding sitagliptin to usual care, as compared with usual care alone, in patients with type 2 diabetes and established cardiovascular disease.	P: 14,671 *P* ≥ 65 years: 7735 P using sitagliptin 100 mg/d (or 50 mg/d based on eGFR^b^): 3813 P using matching placebo: 3816	Median follow-up 3 years	Composite cardiovascular outcome defined as first confirmed event of cardiovascular death, nonfatal myocardial infarction, nonfatal stroke, or hospitalization for unstable angina.
Hartley et al. 2015 [37] (Search 3B)	Randomised, parallel-group, multinational, non-inferiority clinical trial with an active controlled, double-blind treatment period	To evaluate the efficacy and tolerability of sitagliptin compared with glimepiride in elderly patients with type 2 diabetes mellitus and inadequate glycemic control with diet and exercise alone.	P: 480 *P* ≥ 65 years: 480 P using sitagliptin (dose based on eGFR^c^): 241 P using matching placebo or glimepiride (1 mg once daily up to 6 mg/day): 239	30 weeks	Primary safety endpoint: incidence of AEs of symptomatic hypoglycaemia, defined as an episode with clinical symptoms attributed to hypoglycaemia, without regard to glucose level. Asymptomatic hypoglycaemia, defined as episodes without symptoms of hypoglycaemia, but with a glucose level ≤ 70 mg/dL, was also reported.
Kadowaki et al. 2014 [38] (Search 3B)	Randomised, double-blind, placebo-controlled study	To confirm the efficacy and safety, including the risk of hypoglycaemia, of teneligliptin added to glimepiride in Japanese patients with T2DM inadequately controlled with glimepiride monotherapy.	P: 194 *P* ≥ 65 years: 61 P using teneligliptin + glimepiride: 27 P using placebo + glimepiride: 34	12-week randomised double-blind period with teneligliptin 20 mg or placebo. 40-week open-label period with teneligliptin 20 or 40 mg. 2-week period without any study drug.	AEs (included hypoglycaemia events) and ADRs.
Matthews et al. 2010 [55] (Search 3B)	Multicentre, randomised, double-blind, double-dummy, active-controlled study	To show that vildagliptin added to metformin is non-inferior to glimepiride in reducing HbA1c levels from baseline over 2 years.	P: 3118 *P* ≥ 65 years: 789 P using vildagliptin: 392 P using glimepiride: 397	2 years	AEs, SAEs, and hypoglycaemic events.
Rosenstock et al. 2013 [59] (Search 3B)	Multicentre, randomised, double-blind, active controlled study	To prospectively evaluate the efficacy and safety of alogliptin versus glipizide in elderly patients with T2DM over 1 year of treatment.	P: 441 *P* ≥ 65 years: 441 P using alogliptin: 222 P using glipizide: 219	54 weeks	AEs, hypoglycaemia and major cardiac events.
Schernthaner et al. 2015 [39] (Search 3B)	Multinational, randomised, double-blind, phase IIIb/IV study (GENERATION study)	To assess efficacy and safety of adjunctive saxagliptin vs glimepiride in elderly patients with type 2 diabetes and inadequate glycaemic control.	P: 720 *P* ≥ 75 years: 287 P using saxagliptin + metformin: 143 P using glimepiride + metformin: 144	52 weeks	AEs, proportion of patients with ≥1 confirmed/severe hypoglycaemic event.
Schweizer et al. 2009 [40] (Search 3A)	Double-blind, randomised, multicentre, active-controlled, parallel-group study	To evaluate the efficacy and tolerability of DPP-4 inhibitor, vildagliptin and metformin in drug naïve elderly patients with type 2 diabetes.	P: 335 *P* ≥ 65 years: 335 P using vildagliptin: 169 P using metformin: 166	24 weeks	AEs, SAEs, hypoglycaemia and cardiovascular / cerebrovascular events.
Schweizer et al. 2013 [53] (Search 3B)	Post hoc sub-analysis of a multi-centre, randomised, double-blind, parallel-group	To assess the efficacy and tolerability of vildagliptin in elderly T2DM patients with renal impairment.	P: 105 *P* ≥ 75 years: 105 P using vildagliptin: 50 P using placebo: 55	24 weeks	AEs, SAEs and hypoglycaemia.
Scirica et al. 2013 [12] Scirica et al. 2014 [41] Leiter et al. 2015 [42] Mosenzon et al. 2015 [43] (Search 3B)	Multicentre, randomised, double-blind, placebo-controlled trial (Saxagliptin Assessment of Vascular Outcomes Recorded in Patients with Diabetes Mellitus [SAVOR] - Thrombolysis in Myocardial Infarction [TIMI] 53 study)	Scirica et al. 2013 and Scirica et al. 2014 To evaluate the safety and efficacy of saxagliptin with respect to CV outcomes in patients with diabetes mellitus who are at risk for CV events. Leiter et al. 2015 To examine the safety and CV effects of saxagliptin in the predefined elderly (≥ 65 years) and very elderly (≥ 75 years) subpopulations. Mosenzon et al. 2015 To compare the incidence of fractures between patients with saxagliptin and patients with placebo.	P: 16,492 *P* ≥ 65 years: 8561 P using saxagliptin: 4290 P using placebo: 4271 *P* ≥ 75 years: 2330 P using saxagliptin: 1169 P using placebo: 1161	2 years	Scirica et al. 2013 Composite of CV death, MI, or ischemic stroke. Scirica et al. 2014 Hospitalisation for heart failure. Leiter et al. 2015 Primary outcome: composite of CV mortality, nonfatal MI, or nonfatal ischemic stroke. Secondary outcomes: primary composite outcome plus hospitalisation for HF, coronary revascularization, or unstable angina and all components of primary secondary outcomes. Mosenzon et al. 2015 Bone fractures.
Strain et al. 2013 [52] (Search 3B)	Multicentre, randomised, double-blind, placebo-controlled study	To assess the feasibility of setting and achieving investigator-defined individualised treatment targets for a period of 24 weeks in elderly patients with type 2 diabetes (drug-naive or inadequately controlled on oral agents), with the addition of a single oral agent: vildagliptin.	P: 278 *P* ≥ 65 years: 278 P using vildagliptin: 139 P using placebo: 139	24 weeks	AEs, SAEs, and hypoglycaemia.
White et al. 2013 [44] (Search 3B)	Multicenter, randomised, double-blind placebo-controlled trial	To determine whether alogliptin is noninferior to placebo with respect to major cardiovascular events in patients with type 2 diabetes who are at very high cardiovascular risk — those with recent acute coronary syndromes.	P: 5380 *P* ≥ 65 years: 1907 P using alogliptin: 973 P using placebo: 934	Median follow-up 18 months	Composite of death from cardiovascular causes, nonfatal myocardial infarction (MI), or nonfatal stroke. Principal secondary safety end point: primary composite end point with the addition of urgent revascularization due to unstable angina within 24 h after hospital admission
Chang et al. 2015 [33] (Search 3B)	Nationwide retrospective cohort study	To compare CV risks associated with second-line oral antidiabetic agents added to initial metformin therapy.	P: 36,118 Subgroup analysis by age (<65 vs ≥65 years) without number of P in each group P using DPP-4 inhibitors + metformin: 2242 P using SU + metformin: 29,101 P using glinides + metformin: 1553 P using pioglitazone + metformin: 1283 P using α-glucosidase inhibitor + metformin: 1939	Median follow-up ranged from 215 days for the α-glucosidase inhibitor plus metformin group to 305 days for the SU plus metformin group	First hospitalization for acute MI, HF, ischemic stroke after initiation of one of the regimens studied.
Chen et al. 2015 [34] (Search 3B)	Nationwide population-based cohort study	To evaluate efficacy and safety of sitagliptin with respect to cardiovascular outcomes in patients with T2DM and recent ischaemic stroke.	P: 5145 *P* ≥ 75 years: 1435 P using sitagliptin: 486 P using comparison^d^: 949	Mean follow-up 1.17 years (0.75)^e^	Primary outcome: composite event of ischemic stroke, MI, or CV death. Secondary outcomes: haemorrhagic stroke, nonfatal ischemic stroke, nonfatal acute MI, deaths of any cause, and hospitalisation for HF. Safety outcomes: acute or chronic pancreatitis, hypoglycaemia, hyperosmolar hyperglycaemic state, and diabetic ketoacidosis.
Driessen et al. 2014 [45] (Reference list)	Retrospective population based cohort study	To investigate the association between the use of DPP4-I and the risk of fracture.	P: 433,632 *P* ≥ 70 years: 141,322 P using NIAD: 68,801 P without prescription of NIAD: 68,015 P using DPP-4 inhibitor: 4506	Median follow-up: P using NIAD: 3.7 years (1.61–5.22)^f^ P without prescription of NIAD: 3.95 years (1.79–5.22)^f^ P using DPP-4 inhibitor: 5.0 years (2.95–5.16)^f^	Any fracture.
Giorda et al. 2015 [48] (Search 3B)	Population-based nested case-control study	To compare the occurrence of HF in relation to DPP-4 inhibitor use versus any antidiabetic treatment.	Any admission for HF Cases: 14,613 Controls: 146,130 *P* ≥ 65 years: Cases: 13,736 Controls: 137,362 Incident HF Cases: 7212 Controls: 72,120 *P* ≥ 65 years: Cases: 6779 Controls: 67,793 Re-admission for HF Cases: 1712 Controls: 17,222 *P* ≥ 65 years: Cases: 1609 Controls: 16,189 All-cause mortality Cases: 38,248 Controls: 382,313 *P* ≥ 65 years: Cases: 36,335 Controls: 363,197	Not reported	Any admission for HF, incident HF, re-admission for HF, all-cause mortality.
Mistry et al. 2011 [57] (Search 3B)	Retrospective observational survey	To obtain efficacy and safety data on HbA1C levels and incidence of hypoglycaemia in elderly patients who were receiving vildagliptin.	P: 72 *P* ≥ 65 years: 72 P using vildagliptin + metformin: 52 P using vildagliptin +2 OADs (metformin, SUs and/or TZDs): 20	Median follow up: Dual therapy: 7 months Triple therapy: 12 months	Incidence of hypoglycaemic events before and after initiation of vildagliptin.
Ou et al. 2015 [35] (Search 3B)	Nationwide population-based observational cohort study	To compare clinical outcomes of adding DPP-4 inhibitors versus sulfonylureas to metformin therapy in patients with T2DM.	P using DPP-4 inhibitor: 10,089 (propensity score matching) P using SU: 10,089 (propensity score matching) *P* ≥ 65 years: P using DPP-4 inhibitor: 2825 P using SU: 2825	Mean follow-up 3.3 years	All-cause mortality, MACEs (including ischemic stroke and MI), hospitalisation for HF, and hospitalisation for hypoglycaemia.
Penfornis et al. 2012 [49] (Search 3B)	Prospective cohort study	To compare DPP-4 inhibitors with COAD in the real-life treatment of elderly patients with T2DM uncontrolled on metformin alone. The primary objective was to assess the incidence of hypoglycaemic episodes in relationship with glycaemic control assessed by HbA1c level.	P: 1188 *P* ≥ 65 years: 1188 P using DPP-4 inhibitors: 931 P using COAD: 257	6 months	Hypoglycaemic events.
Shih et al. 2015 [36] (Search 3B)	Nested case-control study from a cohort of patients with T2DM treated with OADs	To investigate whether susceptibility to sepsis differed among patients with T2DM taking different classes of OAD.	Cases: 43,015 Controls: 43,015 *P* ≥ 65 years: Cases: 41,725 Controls: 41,725	Not reported	First hospitalisation for sepsis.
Sicras-Mainar and Navarro-Artieda 2014 [50] (Search 3B)	Multicenter, retrospective, observational study	To describe the clinical (treatment adherence, metabolic control, hypoglycemia, and macrovascular complications) and economic (resource use and costs) consequences of using a combination of metformin + vildagliptin to treat type 2 diabetes in elderly patients.	P: 987 *P* ≥ 65 years: 987 P using metformin + vildagliptin: 270 P using metformin + SU: 717	24 months	Hypoglycaemia. Macrovascular complications and cardiovascular events (heart disease, cardiac ischemia, acute myocardial infarction, and heart failure), cerebrovascular disease (stroke [ischemic or haemorrhagic], and transient ischemic attack), all types of peripheral arterial disease and renal disease.
Tziomalos et al. 2015 [51] (Search 3B)	Observational study	To evaluate whether prior antidiabetic treatment affects acute ischaemic stroke severity and in-hospital outcome and whether there are differences between antidiabetic agents regarding these effects.	P: 100 *P* ≥ 65 years: 98 P using DPP-4 inhibitors: 26 P using other antidiabetic agents: 72	Not reported	Acute ischemic stroke severity measured with the modified Rankin Scale score at discharge and with in-hospital mortality.
Viljoen et al. 2013 [46] (Search 3B)	Observational study	To study the efficacy and tolerability of DPP-4 inhibitors in older patients with type 2 diabetes whilst focusing on particular pertinent aspects relevant to care of older persons.	P: 431 *P* ≥ 65 years: 431 P using DPP-4 inhibitors: 129 P never treated with DPP-4 inhibitors: 302	Not reported	Hypoglycaemia.
Yu et al. 2015 [47] (Search 3B)	Cohort study with a nested case-control analysis	To determine whether the use of incretin-based drugs, including DPP-4 inhibitors and GLP-1 analogs, is associated with an increased risk of CHF among patients with T2DM.	P: 57,737 Incident cases of hospitalised CHF: 1118 Matched controls: 17,626 *P* ≥ 65 years: Cases: 861 Controls: 13,572	Mean duration of treated T2DM 2.4 (3.5)^e^ years	Hospitalisation for a first CHF.

*ADRs* Adverse drug reactions, *AEs* Adverse events, *COAD* Conventional oral antidiabetic drugs, *CV* Cardiovascular, *FPG* Fasting plasma glucose, *MACE* Major adverse CV events, *MedDRA* Medical Dictionary for Regulatory Activities, *MI* Myocardial infraction, *NIAD* Non-insulin anti-diabetic drug, *OA* Oral antidiabetics, *OAD* Oral antidiabetic agent, *P* Participants, *PPG* Postprandial plasma glucose, *SAEs* Serious adverse events, *SAP* Stable angina pectoris, *SU* Sulfonylurea, *TIA* Transient ischaemic attacks, *TZD* Thiazolidinedione, *T2DM* Type 2 diabetes mellitus, *UAP* Unstable angina pectoris, * unreported counts were derived from available data where possible

^a^median (minimum, maximum); ^b^ sitagliptin 50 mg daily if the baseline estimated glomerular filtration rate (eGFR) was ≥30 and <50 mL per minute per 1.73 m^2^; ^c^ if baseline eGFR was ≥50 mL per minute per 1.73 m^2^ received sitagliptin 100 mg once daily and if baseline eGFR was ≥35 and <50 mL per minute per 1.73 m^2^ received sitagliptin 50 mg once daily; ^d^ patients who did not receive sitagliptin; ^e^ standard deviation; ^f^ interquartile range

### Study designs

Seventeen of the included studies were interventional designs, one was an MA and 12 were observational in nature. None of the individual studies were also part of the MA. Length of follow-up varied from 12 weeks to 5 years. Data on outcomes was extracted at the end of follow-up for each included study. In 16 out of 30 studies information was given about the countries where studies had been conducted: the USA [29, 30], Australia, Canada, Denmark, the Netherlands and Sweden [31], Taiwan [32–36], 38 countries [13], 13 countries [37], Japan [38], 12 European countries and Mexico [39], 14 European countries [40], 26 countries [12, 41–43], 49 countries [44], UK [45–47], Italy [48], France [49], Spain [50], and Greece [51].

### Participants

Table 1 shows included studies involving older adults (at least 80% people ≥65 years: 16 studies) or presenting subgroup analyses in participants ≥65 years (11 studies including the meta-analysis), ≥75 years (2 studies), and ≥70 years (1 study). Additional file 3: Table S1 shows the characteristics of the participants in the included studies. Age is reported as mean or median years; for the whole sample where available, else for the different treatment groups. Mean age was reported in 27 studies and ranged from 53.1 to 80.2 years. Median age was reported in 3 studies and ranged from 58 to 77 years.

All included studies reported on participant sex (30 studies), though in some cases by treatment group only. The percentage of male participants ranged from 36.7% to 71.6%.

Fourteen studies reported ethnicity with the most common classification being white (range: 53.9 to 98.6%). Information about the care setting was reported by five studies: primary care in the USA [29], primary care in the UK [45], primary care in France [49], hospital department of internal medicine in Greece [51], and primary and hospital care in the UK [47]. Information about comorbidities was provided by 26 out of 30 studies. Concomitant diseases were frequent and hypertension and dyslipidaemia most commonly reported. Eighteen studies reported on concomitant medications with a majority of patients taking antihypertensive and lipid-lowering medications. Frailty status was reported by one study [52], with about 10% of patients assessed as frail. One study reported on disability after stroke as the main outcome, but no baseline data on disability were provided [51]. Cognitive status was not reported by any of the studies.

### Interventions and outcomes

Most of the included studies addressed only our lower tier endpoints: adverse events and hypoglycaemia. A minority of studies investigated Tier 2 outcomes such as death, hospitalisation, cardiovascular events and, in one case, functional status. We found no studies in older people reporting on the clinically relevant endpoints of: quality of life, life expectancy, cognitive impairment or cognitive status.

### Vildagliptin

Vildagliptin (50 or 100 mg/daily) was examined in 9 out of 30 studies. Vildagliptin was compared with placebo in two trials [52, 53], with glimepiride in two trials [54, 55], with metformin in one trial [40] and with thiazolidinediones in one trial [29]. Also included were one uncontrolled trial on vildagliptin [56] and two observational studies [50, 57]. The outcomes for these studies were adverse events [29, 40, 52–56], serious adverse events [52, 54–56], hypoglycaemia [40, 50, 52–57] and a list of other outcomes by Sicras-Mainar and Navarro-Artieda (2014) (macrovascular complications and cardiovascular events, cerebrovascular disease, all types of peripheral arterial disease and renal disease) [50].

### Sitagliptin

Sitagliptin (25 to 100 mg/daily) was examined in five studies: two placebo-controlled [13, 30], one uncontrolled [32], one active controlled [37], and one cohort study [34]. Three of these studies included adverse events as one of their outcomes and their primary endpoint was change in HbA1c [30, 34, 37]. The other two studies reported a composite of cardiovascular events as their primary endpoints [13, 34].

### Linagliptin

Linagliptin (5 mg/daily) was compared with placebo in one trial [31]. The outcomes were adverse events, hypoglycaemia, and cardiovascular events [31]. Also, one meta-analysis investigated the cardiovascular safety of linagliptin [58].

### Teneligliptin

Teneligliptin (20 mg/daily) was compared to placebo in one study [38], with adverse events and hypoglycaemia as outcomes.

### Alogliptin

Alogliptin (25 mg/daily) was compared to glipizide in one study [59] and to placebo in another study [44]. Adverse events and hypoglycaemia were the outcomes in one study [59]. The other study used a composite outcome of death from cardiovascular causes, nonfatal myocardial infarction, or nonfatal stroke [44].

### Saxagliptin

Saxagliptin (5 mg/daily) was compared to placebo in one study reported by four publications with the following outcomes: a composite outcome of cardiovascular death, myocardial infarction, or ischemic stroke [41], hospitalisation for heart failure [12], a composite outcome of cardiovascular mortality, nonfatal myocardial infarction, or nonfatal ischemic stroke with and without hospitalisation for heart failure, coronary revascularization, or unstable angina as well as the individual components [42], and bone fractures [43]. One randomised trial compared saxagliptin (5 mg/daily) against glimepiride (1 mg/daily) with hypoglycaemia and adverse events as safety outcomes and glycaemic control as the primary outcome.

### Any DPP-4 inhibitor

Nine observational studies compared patients treated with DPP-4 inhibitors with patients not receiving DPP-4 inhibitors [46]; other antidiabetic drugs [33, 35, 45, 49, 51]; or between cases and controls [36, 47, 48]. These studies reported on the following outcomes: hypoglycaemia, fractures, disability after stroke (with the modified Rankin scale), cardiovascular events, hospitalisation for heart failure, hospitalisation for sepsis, and mortality.

### Excluded studies

Additional file 1 provides the full list of reasons for exclusion of studies after full text analysis. The main reason for exclusion was that the study population did not match our age criteria for inclusion (*n* = 298).

### Main findings

Twenty-eight studies provided evidence on relevant outcomes comparing DPP-4 inhibitors against an alternative (i.e. non-DPP-4) drug regimen or placebo. For each study and outcome Table 2 summarises the results for the DPP-4 inhibitor and comparison groups, provides estimated risk ratios with 95% confidence intervals, and reports any statistical comparisons from the study itself. To help interpretation, Table 2 organises the results first by Tier of outcome (Tier 1 or Tier 2), and then by form of comparison within Tier (DPP-4 inhibitors versus placebo; versus other active treatments; and as an additional treatment). Two further studies (not tabulated) compared between different DPP-4 inhibitor based-treatments: 1) insulin plus 100 mg vildagliptin versus insulin plus 50 mg vildagliptin dose [56]; 2) vildagliptin plus metformin versus vildagliptin plus 2 oral antidiabetic agents (metformin, sulfonylureas and/or thiazolidinediones) [57]. Quality of studies is also reported in Table 2.

**Table 2:** Summary of study findings

Authors and publication year	Outcomes	DPP-4 inhibitor cases/n^a^ (%)	Comparator cases/n^a^ (%)	Risk ratio^b^ (95% CI)	Reported Statistical comparison	Result favours^c^
Tier 1 outcomes (hypoglycaemia and adverse events), comparisons against placebo						
Barnett et al. 2013 [31] QA^d^ = moderate	SAEs	Linagliptin	Placebo			
		14/162 (8.6)	5/79 (6.3)	1.37 (0.51, 3.66)	NR	C
	Severe AEs	9/162 (5.6)	3/79 (3.8)	1.46 (0.41, 5.25)	NR	C
	Significant AEs	4/162 (2.5)	0/79 (0.0)	4.40 (0.24, 80.8)	NR	C
	Hypoglycaemia	37/162 (22.8)	13/79 (16.5)	1.39 (0.78, 2.46)	NR	C
Barzilai et al. 2011 [30] QA^d^ = moderate		Sitagliptin	Placebo			
	Clinical AEs	47/102 (46.1)	55/104 (52.9)	0.87 (0.59, 1.29)	Diff in % = −6.8%, (−20.0, 6.7)	D
	Clinical SAEs	7/102 (6.9)	14/104 (13.5)	0.51 (0.21, 1.26)	Diff in % = −6.6%, (−15.2, 1.9)	D
	Hypoglycaemia	0/102 (0.0)	0/102 (0.0)	1.0 (0.02, 49.9)	NR	Neither
SAVOR-TIMI 53		Saxagliptin	Placebo			
Mosenzon et al. 2015 [43] Subgroup P > =75 QA^d^ = high	Bone fracture	57/1169	51/1161	1.11 (0.77, 1.61)	HR = 1.13 (0.77, 1.65)	C
Schweizer et al. 2013 [53] QA^d^ = low		Vildagliptin	Placebo			
	AEs	29/50 (58.0)	40/55 (72.7)	0.80 (0.49, 1.29)	NR	D
	SAEs	7/50 (14.0)	9/55 (16.4)	0.86 (0.32, 2.30)	NR	D
	Hypoglycaemia	0.49 events per patient-year	0.96 events per patient-year	0.53 (0.26, 1.08)	*p* = 0.970	D
Shih et al. 2015 [36] QA^d^ = low	Hospitalisation for sepsis:	DPP-4 inhibitor use by cases^e^	DPP-4 inhibitor use by controls^e^			
	Current DPP-4 users only	1148/43015 (2.7)	1152/43015 (2.7)	1.01 (0.93, 1.09)	OR = 0.97 (0.89, 1.07)	D
	Used any time in past year	3523/43015 (8.2)	3276/43015 (7.6)	1.09 (1.03,1.14)	OR = 1.01 (0.95, 1.06)	C
Strain et al. 2013 [52]		Vildagliptin	Placebo			
QA^d^ = high	AEs	66/139 (47.5)	63/139 (45.3)	1.05 (0.81, 1.35)	NR	C
	SAEs	8/139 (5.8)	5/139 (3.6)	1.60 (0.54, 4.77)	NR	C
	Hypoglycaemia	3/139 (2.2)	1/139 (0.7)	3.00 (0.32, 28.5)	NR	C
Tier 1 outcomes (hypoglycaemia and adverse events), comparisons against other active treatments						
Banerji et al. 2010 [29]	Normal renal function	Vildagliptin + metformin	TZD + metformin			
QA^d^ = low	AEs	54/144 (37.5)	29/84 (34.5)	1.09 (0.76, 1.56)	NR	C
Subgroup *P* ≥ 65	SAEs	2/144 (1.4)	1/84 (1.2)	1.17 (0.11, 12.7)	NR	C
	Mildly impaired renal function					
	AEs	59/171 (34.5)	32/77 (41.6)	0.83 (0.59, 1.16)	NR	D
	SAEs	5/171 (2.9)	4/77 (5.2)	0.56 (0.16, 2.04)	NR	D
Ferrannini et al. 2009 [54] QA^d^ = low Subgroup *P* ≥ 65	Hypoglycaemic events	Vildagliptin	Glimepiride			
		6/351 (1.7)	59/361 (16.4)	0.1 (0.05, 0.24)	NR	D
Hartley 2015 [37] QA^d^ = low		Sitagliptin	Glimepiride			
	AEs	118/241 (49.0)	115/236 (48.7)	1.00 (0.84, 1.21)	NR	Neither
	SAEs	7/241 (2.9)	6/236 (2.5)	1.14 (0.39, 3.35)	NR	C
	Asymptomatic hypoglycemia	16/241 (6.6)	35/236 (14.8)	0.45 (0.25, 0.79)	NR	D
	Symptomatic hypoglycemia	2/241 (0.8)	11/236 (4.7)	0.18 (0.04, 0.79)	*p* = 0.009	D
Matthews et al. 2010 [55] QA^d^ = low Subgroup *P* ≥ 65		Vildagliptin	Glimepiride			
	Hypoglycaemia	8/392 (2.1)	69/397 (17.5)	0.12 (0.06, 0.24)	*p* < 0.001	D
Penfornis et al. 2012 [49] QA^d^ = low		DPP-4 inhibitors	COAD			
	Hypoglycaemia	60/931 (6.4)	52/257 (20.1)	0.32 (0.23, 0.45)	*p* < 0.001	D
	Severe hypoglycaemia	1/931 (0.1)	6/257 (2.4)	0.05 (0.01, 0.38)	*p* = 0.001	D
Rosenstock et al. 2013 [59] QA^d^ = low		Alogliptin	Glipizide			
	Hypoglycaemia	12/222 (5.4)	57/219 (26.0)	0.21 (0.11, 0.39)	NR	D
	AEs	163/222 (73.4)	151/219 (68.9)	1.06 (0.85, 1.33)	NR	C
	SAEs	16/222 (7.2)	13/219 (5.9)	1.21 (0.58, 2.52)	NR	C
Schernthaner et al. 2015 [59] QA^d^ = low		Saxagliptin + metformin	Glimepiride + metformin			
	Hypoglycaemia	21/359 (5.8)	125/359 (34.8)	0.17 (0.11, 0.26)	NR	D
	Severe hypoglycaemia	4/359 (1.1)	55/359 (15.3)	0.07 (0.03, 0.20)	OR = 0.06 (0.02, 0.17)	D
	AEs (excluding hypoglycaemia)	213/359 (59.3)	213/359 (59.3)	1.00 (0.89, 1.13)	NR	Neither
	SAEs	41/359 (11.4)	32/359 (8.9)	1.28 (0.83, 1.99)	NR	C
	Deaths	1/359 (0.3)	1/359 (0.3)	1.00 (0.06, 15.93)	NR	Neither
Schweizer et al. 2009 [40] QA^d^ = low		Vildagliptin	Metformin			
	AEs	74/167 (44.3)	83/165 (50.3)	0.88 (0.70, 1.11)	NR	D
	SAEs	5/167 (3.0)	6/165 (3.6)	0.82 (0.26, 2.65)	NR	D
	Gastrointestinal AEs	25/167 (15.0)	41/165 (24.8)	0.60 (0.38, 0.94)	NR	D
	Hypoglycaemia	0/167 (0.0)	2/165 (1.2)	0.20 (0.01, 4.09)	NR	D
Sicras-Mainar and Navarro-Artieda 2014 [50] QA^d^ = very low		Vildagliptin + metformin	Sulfonylureas + metformin			
	Hypoglycaemia	47/270 (17.4)	307/717 (42.8)	0.41 (0.31, 0.53)	*p* < 0.001	D
Viljoen et al. 2013 [46] QA^d^ = very low		DPP-4 inhibitors	Never treated with DPP-4			
	Hypoglycaemia	4/129 (3.1)	24/302 (7.9)	0.39 (0.14, 1.10)	*p* = 0.062	D
Driessen et al. 2014 [45] QA^d^ = low	Fractures	DPP-4 inhibitor	Other non-insulin anti-diabetic drugs			
	70–79 years	NR	NR		HR = 1.16 (0.95, 1.42)	C
	80 + years	NR	NR		HR = 1.0 (0.74,1.34)	Neither
Tier 1 outcomes (hypoglycaemia and adverse events), DPP-4 inhibitors as an additional treatment						
Chien et al. 2011 [32] QA^d^ = low		Sitagliptin + OAD combinations	OAD combinations			
	AEs	5/49 (10.2)	3/49 (6.1)	1.67 (0.40, 6.97)	NR	C
	Hypoglycaemia	1/49 (2.0)	0/49 (0.0)	3.0 (0.13, 71.9)	NR	C
Kadowaki et al. 2014 [38] Subgroup *P* ≥ 65 QA^d^ = low		Teneligliptin + glimepiride	Placebo + glimepiride			
	AEs (including hypoglycaemia)	0/27 (0.0)	1/34 (2.9)	0.42 (0.02, 9.87)	NR	D
	ADRs (including hypoglycaemia)	0/27 (0.0)	1/34 (2.9)	0.42 (0.02, 9.87)	NR	D
Tier 2 outcomes (cardiovascular outcomes), comparisons against placebo						
Johansen et al. 2012 [58] QA^d^ = low Subgroup *P* ≥ 65		Linagliptin	Comparators^f^			
	Fatal or non-fatal MI or stroke, or hospitalisation for unstable angina pectoris	5/929 (0.5)	14/549 (2.6)	0.21 (0.08, 0.58)	HR = 0.28, (0.1–0.79)	D
TECOS Green et al. 2015 [13] QA^d^ = low Subgroup *P* ≥ 65		Sitagliptin	Placebo			
	Composite CV outcome (first confirmed event of CV death, non-fatal MI, nonfatal stroke, or hospitalization for unstable angina)	NR	NR		HR = 1.01 (0.90, 1.15)	C
SAVOR-TIMI 53 Scirica et al. 2013 [12] Scirica et al. 2014 [41] Subgroup *P* ≥ 75 Leiter et al. 2015 [42] Subgroup *P* ≥ 65 QA^d^ = high	Subgroup *P* ≥ 75	Saxagliptin	Placebo			
	CV death, nonfatal MI, or nonfatal ischemic stroke	117/1169 (10.0)	129/1161 (11.3)	0.90 (0.71, 1.14)	HR = 0.96 (0.75, 1.22)	D
	Hospitalisation for HF Subgroup *P* ≥ 65	79/1169 (6.8)	57/1161 (4.9)	1.38 (0.99, 1.92)	HR = 1.47 (1.05, 2.08)	C
	CV death, nonfatal MI, or nonfatal ischemic stroke	334/4290 (7.8)	367/4271(8.6)	0.91 (0.79, 1.04)	HR = 0.92 (0.79, 1.06)	D
	CV death, MI, stroke, hospitalization for unstable angina, HF, or coronary revascularization	570/4290 (13.3)	593/4271(13.9)	0.96 (0.86, 1.06)	HR = 0.96 (0.85, 1.07)	D
	MI	141/4290 (3.3)	170/4271(4.0)	0.83 (0.66, 1.03)	HR = 0.86 (0.69, 1.07)	D
	CV mortality	158/4290 (3.7)	166/4271(3.9)	0.95 (0.77, 1.17)	HR = 0.92 (0.74, 1.13)	D
	Non-CV mortality	98/4290 (2.3)	76/4271(1.8)	1.28 (0.95, 1.73)	HR = 1.22 (0.92, 1.63)	C
	All-cause mortality	253/4290 (5.9)	239/4271(5.6)	1.05 (0.89, 1.25)	HR = 1.01 (0.86, 1.20)	C
	Nonfatal ischemic stroke	77/4290 (1.8)	68/4271(1.6)	1.13 (0.82, 1.56)	HR = 1.17 (0.85, 1.61)	C
	Hospitalisation for/due to:					
	CR	210/4290 (4.9)	234/4271(5.5)	0.89 (0.75, 1.07)	HR = 0.87 (0.73, 1.05)	D
	HF	180/4290 (4.2)	149/4271(3.5)	1.20 (0.97, 1.49)	HR = 1.25 (1.01, 1.56)	C
	Hypoglycaemia	34/4290 (0.8)	25/4271(0.6)	1.35 (0.81, 2.27)	HR = 1.29 (0.78, 2.14)	C
	Unstable angina	38/4290 (0.9)	38/4271(0.9)	1.00 (0.64, 1.56)	HR = 0.89 (0.56, 1.39)	D
White et al. 2013 [44] QA^d^ = low		Alogliptin	Placebo			
	Death from CV causes, or nonfatal MI or stroke	141/934 (15.1)	149/973 (15.3)	0.99 (0.8, 1.22)	HR = 0.98 (0.78, 1.24)	D
Tier 2 outcomes (cardiovascular outcomes), comparisons against other active treatments						
Chang et al. 2015 [33] Subgroup *P* ≥ 65 QA^d^ = low		DPP-4 inhibitors plus metformin	Sulfonylureas plus metformin			
	Any CV event	NR	NR		HR = 0.86 (0.72, 1.02)	D
	MI	NR	NR		HR = 0.86 (0.44, 1.70)	D
	HF	NR	NR		HR = 1.01 (0.72, 1.43)	C
	Ischaemic stroke	NR	NR		HR = 0.83 (0.68, 1.02)	D
Chen et al. 2015 [34] Subgroup *P* ≥ 75 QA^d^ = low		Sitagliptin	Non-sitagliptin			
	Composite of ischemic stroke, MI, or CV death	59/486 (12.1)	104/949 (11.0)	1.11 (0.82, 1.50)	*p* = 0.463	C
	Ischemic stroke	42/486 (8.6)	77/949 (8.1)	1.07 (0.74, 1.53)	*p* = 0.705	C
Giorda et al. 2015 [48] QA^d^ = low		DPP-4 inhibitor use by cases^e^	DPP-4 inhibitor use by controls^e^			
	Any admission for HF	256/14613 (1.8)	2881/146130 (2.0)	0.89 (0.78, 1.01)	OR = 1.00 (0.94, 1.07)	Neither
	Incident HF	135/7212 (1.9)	1285/72120 (1.8)	1.05 (0.88, 1.25)	OR = 1.01 (0.92, 1.11)	C
	Re-admission for HF	37/1727 (2.1)	338/17222 (2.0)	1.09 (0.78, 1.53)	OR = 1.02 (0.84, 1.22)	C
	All-cause mortality	306/38248 (0.8)	6717/382313 (1.8)	0.46 (0.41, 0.51)	OR = 0.94 (0.90, 0.98)	D
Ou et al. 2015 [35] Subgroup P 61–80 Subgroup *P* ≥ 81 QA^d^ = low		DPP-4 inhibitors + metformin	Sulfonylureas + metformin			
	All-cause mortality					
	61–80	NR	NR		HR = 0.57 (0.46, 0.71)	D
	*P* ≥ 81	NR	NR		HR = 0.61 (0.43, 0.87)	D
	MI					
	61–80	NR	NR		HR = 0.47 (0.26, 0.83)	D
	*P* ≥ 81	NR	NR		HR = 0.70 (0.25, 2.00)	D
	Ischemic stroke					
	61–80	NR	NR		HR = 0.49 (0.24, 1.00)	D
	*P* ≥ 81	NR	NR		HR = 0.63 (0.50, 0.80)	D
	Hospitalisation for HF					
	61–80	NR	NR		HR = 0.78 (0.52, 1.16)	D
	*P* ≥ 81	NR	NR		HR = 0.33 (0.13, 0.87)	D
Rosenstock et al. 2013 [59] QA^d^ = low		Alogliptin	Glipizide			
	Major adverse cardiac events	1/222 (0.5)	2/219 (0.9)	0.49 (0.04, 5.44)	NR	D
Schweizer et al. 2009 [40] QA^d^ = low		Vildagliptin	Metformin			
	CV and cerebrovascular events	2/167 (1.2)	2/165 (1.2)	1.0 (0.14, 6.93)	NR	Neither
Sicras-Mainar and Navarro-Artieda 2014 [50] QA^d^ = very low		Vildagliptin + metformin	Sulfonylureas + metformin			
	CV events	12/270 (4.4)	62/717 (8.6)	0.51 (0.28, 0.94)	*p* = 0.025	D
	Ischemic heart disease	2/270 (0.7)	15/717 (2.1)	0.35 (0.08, 1.54)	*p* = 0.043	D
	Cerebrovascular accident	6/270 (2.2)	31/717 (4.3)	0.51 (0.22, 1.22)	*p* = 0.042	D
	Renal failure	4/270 (1.5)	16/717 (2.2)	0.66 (0.22, 1.97)	*p* = 0.138	D
Tziomalos et al. 2015 [51] QA^d^ = very low		DPP-4 inhibitors	Other antidiabetic drugs			
	In-hospital mortality in people admitted with acute ischaemic stroke	0/27 (0.0)	11/73 (15.1)	0.12 (0.01, 1.91)	*p* < 0.05	D
	Modified Rankin Scale of disability [mean (SD)]	2.1 (1.9)	3.2 (2.1)		*p* < 0.05	D
Yu et al. 2015 [47] QA^d^ = low		DPP-4 inhibitor use by cases^e^	DPP-4 inhibitor use by controls^e^			
	Hospitalisation for HF	54/1118 (4.8)	808/17626 (4.6)	1.05 (0.81, 1.38)	OR = 0.88 (0.63, 1.22)	D

*AEs* Adverse events, *ADRs* Adverse drug reactions, *C* Comparator, *CI* Confidence interval, *COAD* Conventional oral antidiabetic drugs, *CV* Cardiovascular, *D* DPP-4 inhibitor, *Diff* Difference, *HF* Heart failure, *HR* Hazard ratio, *MI* Myocardial Infarction, *CR* Coronary revascularization, *NR* Not Reported, *OAD* Oral anti-diabetic agents, *P* Participants, *SAEs* Serious adverse events, ^a^number of patients with the outcome/total patients, unless stated otherwise; ^b^Zero cell adjustment applied where relevant; ^c^Based on reported comparison or if not reported, the computed risk ratio; ^d^ QA: quality appraisal based on study limitations suggested by Guyatt et al. (2008) [26]; ^e^ Case-control study: cases are patients with the outcome, controls are matched patients without, numerator is count of patients using DPP-4 inhibitors;﻿ ^f^Data pooled over 8 trials, 6 comparing against placebo only

### Comparisons between DPP-4 inhibitors and other drug regimens or placebo

Nineteen studies provided evidence on Tier 1 outcomes. Studies varied in what they classified as adverse events, and as serious, severe, or significant adverse events. Hypoglycaemia was defined by 3 studies as hypoglycaemic symptoms confirmed by self-monitoring of blood glucose <3.1 mmol/l [29, 40, 52]; another study defined symptomatic hypoglycaemia as an episode with clinical symptoms without regard to glucose level, asymptomatic hypoglycaemia was defined as an episode of glucose level ≤ 70 mg/dL without symptoms [37]; another study defined confirmed hypoglycaemia as a symptomatic or asymptomatic event with plasma glucose <3.0 mmol/l without requiring external assistance, severe hypoglycaemia was defined as symptomatic event requiring external assistance without regard to plasma glucose level [39]; the other 3 studies did not provide a definition of hypoglycaemia. In 10 studies [37, 39, 40, 46, 49, 50, 53–55, 59] hypoglycaemia was considerably less frequent in older people treated with DPP-4 inhibitors than in older people on other treatments, on placebo, or when used as an additional medication.

Eleven studies [29–32, 37–40, 52, 53, 59] reported on adverse events other than hypoglycaemia. All showed only small, non-significant, differences. Two studies reported on fractures, one an RCT comparing saxagliptin to placebo [43] and the other a retrospective cohort study comparing DPP-4 inhibitors to other non-insulin anti-diabetic drugs [45]; both finding no significant difference. A nested case-control study reported that hospitalisation for sepsis was not significantly different between cases and controls using DPP-4 inhibitors [36].

Thirteen studies considered Tier 2 endpoints. The results for these generally more impactful outcomes were much more variable. The meta-analysis by Johansen et al. (2012) found that major cardiovascular events (fatal or non-fatal myocardial infarction or stroke, or hospitalisation for unstable angina pectoris) were significantly reduced by around 70% with linagliptin compared to comparators (mostly patients on placebo, but including a minority on anti-diabetic drugs) [58]. However, the large-scale RCTs [12, 13, 42, 44] reported no significant difference between DPP4 inhibitors (sitagliptin, saxagliptin, and alogliptin, respectively) and placebo using a similar endpoint; while one of them found a statistically significant 47% higher risk of hospitalisation for heart failure in the saxagliptin group [41]. Four observational studies reported no significant differences between DPP-4 inhibitors and other active treatments for various cardiovascular outcomes such as myocardial infarction, heart failure, ischaemic stroke, and hospitalisation for heart failure [33, 34, 47, 48], although in one study all-cause mortality was significantly lower in users of DPP-4 inhibitors than in controls [48]. One retrospective observational study reported significantly lower percentages of cardiovascular events, ischemic heart disease, and cerebrovascular accident with vildagliptin plus metformin compared to sulfonylureas plus metformin [50]; but Schweizer et al. (2009) found no difference between vildagliptin and metformin in rates of cardiovascular and cerebrovascular events [40], and Rosenstock et al. (2013) observed that alogliptin and glipizide did not differ in major cardiac events, though in both of these latter randomised trials samples sizes were small and events rare [59]. A cohort study reported significantly less mortality, myocardial infarction, ischemic stroke and hospitalisation for heart failure with DPP-4 inhibitors plus metformin compared to sulfonylureas plus metformin [35]. A small observational study reported significantly lower in-hospital mortality in people admitted with acute ischemic stroke and better scores on the modified Rankin scale of disability with DPP-4 inhibitors compared to other antidiabetics [51].

We deemed study synthesis by meta-analysis inappropriate, due to high heterogeneity of treatments, outcome definitions and follow-up periods. However, to gain a global overview and aid interpretation, Table 2 indicates the treatment arm favoured on each outcome in each study, based purely on the reported direction of effect or (where missing) risk ratio point estimate and ignoring statistical significance. Under this “vote counting” method [19], for the Tier 1 outcomes 8 studies fully favoured the DPP-4 inhibitor, 4 fully favoured the comparator, and 7 were mixed or neutral. For the Tier 2 outcomes, 7 studies favoured DPP-4 inhibitors, 2 favoured comparators, and 4 were mixed or neutral. However, only 2 studies received high overall ratings for quality (see below); both reporting Tier 1 outcomes favouring placebo and one reporting Tier 2 outcomes with mixed results. There was no suggestion for either tier of outcomes of the pattern of results differing according to whether the comparison was a placebo, other active treatment, or DPP-4 inhibitors as an additional treatment.

### Comparisons between DPP-4 inhibitor-based treatments

Both studies reported that hypoglycaemic events were similar between the groups: 1) 2.1 events per patient-year with insulin plus vildagliptin 100 mg versus 2.3 events per patient-year with insulin plus vildagliptin 50 mg [56]; 2) no events with vildagliptin plus metformin versus 1 event with vildagliptin plus 2 oral antidiabetic agents [57].

### Quality appraisal of included studies

#### Meta-analysis

One meta-analysis was included [58] and it met 4 out of 11 criteria of the AMSTAR tool (Table 3).

**Table 3:** Quality appraisal for systematic reviews/meta-analysis

Source	Type of study	1. ‘a priori’ design	2. Duplicate selection and data extraction	3. Comprehensive search	4. Status of publication	5. List of studies	6. Characteristics provided	7. Scientific quality assessed	8. Scientific quality in conclusions	9. Methods to combine findings	10. Publication bias	11. Conflict of interest
Johansen et al. 2012 [58]	Meta-analysis	N	U	U	N	Y	Y	N	N	Y	N	Y

*Y* Yes, *N* No, *U* Unclear

#### Clinical trials

Seventeen clinical trials were included and their quality appraisal is shown in Table 4. Only two studies had a low risk of bias for all seven items [12, 41–43, 52]. Four RCTs had reasonable good quality with low risk of selection, performance, and attrition bias [13, 30, 31, 37]. Most studies had low risk of attrition bias. Twelve studies were at high risk of “other bias”. One study was a retrospective analysis of an open-label clinical trial and had a high risk of selection, performance and detection bias [29]. Most of the included clinical trials did not provide enough information to fully assess their risk of bias and had “unclear risk of bias” for at least four of the items.

**Table 4:** Quality appraisal for intervention studies

Source	Type of study	Selection bias		Performance bias	Detection bias	Attrition bias	Reporting bias	
		1. Random sequence generation	2. Allocation concealment	3. Blinding of participants and personnel	4. Blinding of outcome assessment	5. Incomplete outcome data	6. Selective reporting	7. Other bias
Banerji et al. 2010 [29]	Retrospective analysis of the GALIANT study which is a multicenter, randomised, open-label study	LR	HR	HR	HR	LR	UR	HR
Barnett et al. 2013 [31]	Randomised, double-blind, placebo-controlled trial	LR	LR	LR	UR	LR	LR	UR
Barzilai et al. 2011 [30]	Randomised, double-blind, placebo-controlled trial	LR	LR	LR	UR	LR	UR	UR
Chien et al. 2011 [32]	Randomised, open-labelled, parallel-group study	UR	UR	UR	HR	UR	UR	HR
Ferrannini et al. 2009 [54]	Multicentre, randomised, double-blind, active-controlled study	UR	UR	UR	UR	LR	UR	HR
Fonseca et al. 2008 [56]	Multicentre, double-blind, parallel-group, randomised study	UR	UR	UR	UR	LR	UR	HR
Green et al. 2015 [13]	Randomised, double-blind, placebo-controlled study	LR	LR	LR	LR	LR	LR	HR
Hartley et al. 2015 [37]	Randomised, parallel-group, multinational, non-inferiority clinical trial with an active controlled, double-blind treatment period	LR	LR	LR	UR	LR	LR	HR
Kadowaki et al. 2014 [38]	Randomised, double-blind, placebo-controlled study	UR	UR	LR	UR	LR	UR	HR
Matthews et al. 2010 [55]	Multicentre, randomised, double-blind, double-dummy, active-controlled study	UR	UR	LR	UR	LR	UR	HR
Rosenstock et al. 2013 [59]	Multicentre, randomised, double-blind, active controlled study	UR	UR	LR	UR	LR	UR	HR
Schernthaner et al. 2015 [39]	Multinational, randomised, double-blind, phase IIIb/IV study	LR	UR	UR	UR	HR	UR	HR
Schweizer et al. 2009 [40]	Double-blind, randomised, multicentre, active-controlled, parallel-group study	UR	UR	UR	UR	LR	UR	HR
Schweizer et al. 2013 [53]	Post-hoc sub-analysis of a multi-centre, randomised, double-blind, parallel-group	UR	UR	UR	UR	UR	UR	UR
Scirica et al. 2013 [12] Scirica et al. 2014 [41] Leiter et al. 2015 [42] Mosenzon et al. 2015 [43]	Multicentre, randomised, double-blind, placebo-controlled trial	LR	LR	LR	LR	LR	LR	LR
Strain et al. 2013 [52]	Multinational, double-blind, randomised, placebo-controlled	LR	LR	LR	LR	LR	LR	LR
White et al. 2013 [44]	Multicentre, randomised, double-blind placebo-controlled trial	LR	UR	LR	UR	LR	LR	HR

*LR* Low risk of bias, *HR* High risk of bias, *UR* Unclear risk of bias

#### Observational studies

Quality appraisal of the 12 observational studies is shown in Table 5. Six studies reported insufficiently on most of the CASP items to be considered of high quality [36, 46, 49, 50, 57]. The other six studies reported information on most of the CASP items to be considered of high quality [33–35, 45, 47, 48].

**Table 5:** Quality appraisal for observational studies

Source	Type of study	1. Focused issue	2. Appropriate method	3. Recruitment	4. Selection of controls	5. Exposure measured	6. Outcome measured	7. Identified confounding factors	8. Confounding factors in design/analysis	9. Follow up complete	10. Follow up long	11. Results	12. Precise results	13. Believe results	14. Results be applied	15. Results fit evidence
Chang et al. 2015 [33]	Cohort study	Y	Y	Y	NA	Y	Y	Y	Y	Y	Y			Y	Y	N
Chen et al. 2015 [34]	Cohort study	Y	Y	Y	NA	Y	Y	Y	Y	Y	Y			Y	Y	N
Driessen et al. 2014 [45]	Retrospective population based cohort study	Y	Y	Y	NA	Y	Y	Y	Y	Y	Y			Y	Y	N
Giorda et al. 2015 [48]	Case-control study	Y	Y	Y	Y	Y	Y	Y	Y	Y	U			Y	Y	N
Mistry et al. 2011 [57]	retrospective observational survey	Y	U	Y	NA	U	Y	U	U	NA	NA			Y	Y	Y
Ou et al. 2015 [35]	Cohort study	Y	Y	Y	NA	Y	Y	Y	Y	Y	Y			Y	Y	N
Penfornis et al. 2012 [49]	Prospective cohort study	Y	Y	Y	N	U	Y	U	U	Y	Y			Y	U	Y
Shih et al. 2015 [36]	Nested case-control study	Y	Y	Y	Y	Y	Y	U	Y	NA	NA			U	U	N
Sicras-Mainar and Navarro-Artieda 2014 [50]	Retrospective longitudinal study	Y	U	U	U	Y	U	U	N	U	U			U	U	U
Tziomalos et al. 2015 [51]	Observational study	Y	Y	U	NA	Y	Y	U	U	NA	NA			U	U	N
Viljoen et al. 2013 [46]	Observational study	Y	U	N	U	U	U	U	U	NA	NA			Y	Y	Y
Yu et al. 2015 [47]	Cohort study with a nested case-control analysis	Y	N	Y	Y	Y	Y	N	Y	NA	NA			Y	Y	N

*Y* Yes, *N* No, *U* Unclear, *NA* Not applicable. Columns of items 11 and 12 are empty because these can not be answered with Y, N or U

#### Overall ratings of quality

The single meta-analysis was rated as low quality overall because this investigation did not assess the scientific quality of its included studies. The majority of individual studies were rated low or very low overall quality, and only two received a high overall quality rating.

### Involvement of pharmaceutical companies in studies

Twenty-two studies were funded by pharmaceutical companies and authored or co-authored by employees of the sponsor (22 out of 30, 73%). In the remaining eight studies, one study gave no information about funding although authors stated globally they had no conflict of interest [32]; two studies reported no funding and no conflict of interest [33, 51]; two studies reported funding outside pharmaceuticals and no conflict of interest [36, 48]; two studies reported funding outside pharmaceuticals and conflict of interest from some of the authors receiving fees from pharmaceuticals [34, 47]; one study reported no funding but one author declared receiving fees from pharmaceuticals [35]. The 30 included studies were authored by 219 authors: 29% (63 out of 219) were employees of the pharmaceutical sponsor, and an additional 27% declared conflicts of interest (60 out of 219, often consulting fees by the sponsor). Sixty-one authors of 11 publications (61 out of 219, 28%) declared no conflicts of interest. Support by professional medical writers was given in at least 8 publications (8 out of 30, 27%).

### Additional references of interest for the development of recommendations

We found four additional references that were taken into consideration for the development of the recommendation: 1 meta-analysis, 1 pooled analysis, 1 observational study, and a report from the FDA. These are shown in Additional file 4: Table S2. These references did not meet our age or study design criteria for inclusion. They were counted as being relevant to recommendations principally because they provided information about clinically relevant endpoints not adequately addressed by the 30 included studies, albeit for younger populations.

One of the additional references reported that there were no statistically significant differences between vildagliptin compared to other anti-diabetic treatments or placebo for long-term outcomes including acute coronary syndrome, transient ischaemic attack, stroke, myocardial infarction, cardiovascular and cerebrovascular death [60]. Two of the additional references reported an increase in the risk of hospitalisations for heart failure and an increase in heart failure outcomes in people under DDP-4 inhibitors compared to people under other anti-diabetic treatments or placebo [61, 62]. These studies concur with that of Scirica et al. (2013) [12] for patients age 75 and over, included in the present SR.

Furthermore, the U.S. Food and Drug Administration (FDA) and the European Medicines Agency (EMA) reported that there are still some uncertainties with respect to long-term pancreatic safety with DPP-4 inhibitors and evaluation of these outcomes is ongoing [63]. Although the currently available data are reassuring, pancreatitis will continue to be considered a risk associated with these drugs until more data are available. These additional references suggest that certain risks like heart failure and related hospitalisation, and pancreatitis, may be increased with the use of DPP-4 inhibitors compared to other anti-diabetic treatments, independently of age group.

### Recommendations

Recommendations were developed following a standardized schema and reflecting the strength and the quality of the evidence. Two meetings were held by GS (researcher and clinician), YVM (researcher) and ARG (researcher and geriatrician), with AS participating in one of these as a senior clinician and researcher. Subsequent to these meetings we agreed a recommendation which was later confirmed with IK and MMV for its inclusion in the Comprehensive Medication Review (CMR) tool developed within the PRIMA-eDS project.

From the results of our SR and the additional references of interest we developed one recommendation in relation to DPP-4 inhibitors use in older people with type 2 diabetes. The recommendation is that the clinician should consider discontinuing gliptins where the patient has HbA1c < 8.5%, principally because of the sparse and inconsistent evidence for clinically relevant benefits, but taking the patient’s symptoms into account (Table 6). The recommendation was considered as a weak recommendation. The quality was downgraded from high to moderate for indirectness.

**Table 6:** Recommendation for DPP-4 inhibitors in older people with type 2 diabetes

Recommendation	Strength of the recommendation	Quality of the evidence
The patient is taking DPP-4 inhibitors and HbA1c is <8.5% (70 mmol/mol). Please reconsider the use of gliptins for the management of type 2 diabetes in older adults because of scarce data on clinically relevant benefits of their use. Please take the patient’s symptoms into consideration.	Weak Reason: No trial data supporting long-term clinically-relevant benefits in older people. One RCT pointing at possible adverse long-term effects independently from age.	The evidence was graded low quality. It was considered to downgrade the quality of the evidence to low quality because there were study limitations (1 observational study and a pooled analysis), indirectness (most of the studies did not report data in older people, apart from the pooled analysis), inconsistency (different types of DPP-4 inhibitors evaluated), and lack of data of long-term benefits under DPP-4 inhibitors in older people.

We considered glycaemic control in the recommendation although it was not one of our study endpoints. The aim was that clinicians would focus on those patients who may benefit more from the recommendation as they could be already having acceptable glycaemic control. In older people, rigid glycaemic control (<HbA1c 8.0%) has been found to be associated with a higher risk of hypoglycaemia and undesirable long-term outcomes like increased mortality [64]. The target population in the PRIMA-eDS trial were people 75 years or older with at least eight prescribed medications reflecting a high comorbidity burden. An HbA1c <8.5% has been recommended in guidelines as a target goal in older people who have comorbidities, poor health, dementia, frailty or limited life expectancy [8, 65–67]. As a general rule, PRIMA-eDS recommends clinicians to take symptoms into consideration as well as the individual participant characteristics such as frailty level and comorbidities.

## Discussion

Thirty studies reported in 33 publications (one MA, 17 interventional studies and 12 observational studies) were identified which evaluated the use of gliptins for the management of type 2 diabetes in older people and reported on clinically relevant outcomes. While the majority of the studies reported participant data on comorbidities, only one presented data on frailty status. In terms of outcomes, most of the included studies reported on adverse events and hypoglycaemia. Fourteen studies reported on cardiovascular events (such as heart failure, myocardial infarction, and stroke), hospitalisation for heart failure, functional status, cardiovascular mortality, and all-cause mortality either as individual outcomes or combined into a composite outcome. None of these studies evaluated all-cause hospitalisation, quality of life or cognitive status.

In general, studies of DPP-4 inhibitors have shown similar or better safety than placebo and other antidiabetic drugs in older adults with type 2 diabetes, but these safety data are mainly based on short-term outcomes like hypoglycaemic events and acute adverse events. The evidence for longer-term health or quality-of-life benefits is more limited and quite inconsistent, with some studies showing benefits and others increased risks, particularly when the evidence from younger age groups is factored in. In addition, only six studies had reasonably good quality and the results from these provided very little evidence for a benefit in older people from treatment with DPP-4 inhibitors.

DPP-4 inhibitors have been recommended as second line drugs for the management of type 2 diabetes in older adults by several expert groups [68] because of their lower risk of hypoglycaemia. Hypoglycaemia may be very relevant in older people, especially if it is symptomatic and has consequences such as falls. Unfortunately, information on the consequences of the hypoglycaemic events was not clearly reported in most studies. However, the majority of studies included patients with a mean HbA1c ≤8% at baseline. According to current guidelines, for older patients with these HbA1c levels further treatment may not be recommended, especially in those with functional impairment [8, 10, 69]. Rigid glycaemic control beyond an HbA1c of 8% achieved by antidiabetic drugs may be associated with a higher risk of hypoglycaemia and undesirable long-term outcome like increased mortality [64]. At present, it is unclear if the decreased risk for hypoglycaemia seen with the use of gliptins would also be seen in populations with less tight glycaemic control. In terms of effectiveness, we did not use glycaemic control as a clinically relevant endpoint. Glycaemic control has often been regarded as a surrogate endpoint without evidence for a direct relationship to longer term outcomes [70]. However, our omission of evidence for good glycaemic control with less adverse events might have introduced a limitation to our systematic review.

Unfortunately, most studies included in this publication did not provide any information on the frailty level and cognitive status of their participants. Data on some comorbidities and the use of some concomitant drugs were provided but the number of drugs used and concomitant diseases were rarely reported. This limits the interpretation of the results and their applicability to the heterogeneous older population.

Furthermore, with the exception of five studies [32, 33, 36, 48, 51], the rest of the included studies on DPP-4 inhibitors in older people were sponsored by pharmaceutical companies, authored or co-authored by company employees, or included authors working closely with the pharmaceutical sponsor and receiving consultancy or advisory fees (60/219, 28%). A close affiliation between pharmaceutical companies and researchers appears to be a general problem in diabetes research [71] and raises concerns about the independence and integrity of the evidence base for the treatment of diabetes.

Five studies were from Taiwan including a randomised trial [32] and four observational studies [33–36]. All of these observational studies used the same database from the Taiwan National Health Insurance. Although it seems that populations are different in each of these studies, three of them were on DPP-4 inhibitors [33, 35, 36]. It might have been possible that some of the samples included similar populations.

We conducted this SR following an adaption of the standard methodology recommended by the Cochrane collaboration and the PRISMA statement. The searches were conducted in six biomedical literature databases. We developed a stepwise search as part of the methodology for this systematic review and included existing systematic reviews and meta-analyses, as well as individual studies. However, this methodology has not been independently validated. Search 3B was limited to publications since 2005 [21]. Although it is conceivable that some pre-2005 studies were missed in this process, we believe that earlier relevant studies were captured during search 3A, checking of references lists, or snowballing. Furthermore, the first DPP-4 inhibitor (i.e. sitagliptin) was only approved by the FDA in 2006 which gives us confidence that we did not miss relevant studies [72]. Many patients are prescribed combinations of antidiabetic medications and we have not attempted to modulate our recommendation according to the particular treatments being used alongside DPP-4 inhibitors or when these are the sole treatment. This is an important but complex issue that could not be addressed within the objectives of this systematic review, and when re-considering the use of DPP-4 inhibitors with an individual patient, the clinician must take into account any additional treatments the patient may be receiving for their diabetes.

The recommendation derived from the results of this SR aims at increasing awareness in clinicians about the evidence (and lack of evidence) with regard to the use of DPP-4 inhibitors for the management of type 2 diabetes in older adults. Decisions on the prescription or de-prescription of DPP-4 inhibitors should be made taking the symptoms and individual characteristics of each patient into account, including any other antidiabetic medications the patient may be taking, and involving the older person themselves in the decision-making process [73]. HbA1c levels should also be taken into consideration as current guidelines recommend no further treatment in older people with functional impairment and HbA1c <8% [8, 10, 69]. We developed the recommendation based on the results of this SR and the four additional references (studies without age or study design criteria to be included) which provided information about clinically relevant endpoints not adequately addressed by the 30 included studies. It should be noted that the included studies in this SR only provided evidence of a suspected effect on hospitalisations for heart failure with saxagliptin [41, 42]. However, additional references suggest that certain risks like heart failure and related hospitalisation, and pancreatitis, may be increased with the use of DPP-4 inhibitors compared to other anti-diabetic treatments, independently of age group [60–63].

The results of this SR show that further research is needed on the clinically relevant short and long-term risks and benefits of the use of DPP-4 inhibitors for the management of type 2 diabetes in older adults. Older adults living in different settings including care homes, with comorbidities, polypharmacy, cognitive and functional limitations should be represented in the studies.

## Conclusions

Evidence for beneficial clinically relevant outcomes regarding the usage of DPP-4 inhibitors in older people with type 2 diabetes is ambiguous at best. DPP-4 inhibitors appear to be safer compared to other anti-diabetic medications to treat older people with type 2 diabetes. However, these safety data are based only on short-term surrogate outcomes and standard HbA1c control targets, and the characteristics of the studied older people in terms of frailty and medical complexity are not described. In addition, there is a lack of studies independent of pharmaceutical company sponsorship. Independently from age, an increased risk of heart failure outcomes in adults under DDP-4 inhibitors has been reported [74]. Therefore, at present, DPP-4 inhibitors should be prescribed with caution in older patients with type 2 diabetes, especially if HbA1c is already in the therapeutic range of <8.5% recommended by experts for frail older people (from expert-based recommendations).

## Additional files

Additional file 1: Excluded studies. List of excluded studies after full-text check with reasons for exclusion. (XLSX 47 kb)

Additional file 2: Search strategy. Full search terms for each search (searches 1, 2 and 3B). (DOCX 185 kb)

Additional file 3: Participant characteristics. Characteristics of the participants in the included studies. (DOCX 60 kb)

Additional file 4: Additional evidence for recommendation. Additional evidence for recommendation. (DOCX 15 kb)

## Abbreviations

AMSTAR: A Measurement Tool to Assess Systematic Reviews
CASP: Critical Appraisal Skills Programme
CMR: Comprehensive Medication Review
DARE: Database of Abstracts or Reviews of Effects
GRADE: Grading of Recommendations Assessment, Development and Evaluation
HTA: Health Technology Assessment
IPA: International Pharmaceutical Abstracts
PICOS: Population, intervention, comparison, outcomes and study design
PRIMA-eDS: Polypharmacy in chronic diseases: Reduction of Inappropriate Medication and Adverse drug events in elderly populations by electronic Decision Support
PRISMA: Preferred Reporting Items for Systematic Reviews and Meta-Analyses
RCTs: Randomised controlled trials
SR: Systematic review
