# Supplementary material for: Effectiveness and safety of dipeptidyl peptidase 4 inhibitors in the management of type 2 diabetes in older adults: a systematic review and development of recommendations to reduce inappropriate prescribing
Source: BMC Geriatr. 2017 Oct 16;17(Suppl 1):226. doi: 10.1186/s12877-017-0571-8 (PMC5647559; doi:10.1186/s12877-017-0571-8)
Supplement: Supplementary file 2 — Search strategy. Full search terms for each search (searches 1, 2 and 3B). (DOCX 185 kb) [file 12877_2017_571_MOESM2_ESM.docx]

**Dipeptidyl Peptidase 4 Inhibitors in the management of Type 2 Diabetes**

**Search 1**

**Databases**

**EBM Reviews - Cochrane Database of Systematic Reviews**

**EBM Reviews - Database of Abstracts of Reviews of Effects**

|  | |  |  | |
| --- | --- | --- | --- | --- |
| **Population** | | | | |
|  | geriatrics.mp. or exp geriatrics/ | | |  |
|  | geriatric patient.mp. | | |  |
|  | geriatric*.mp. | | |  |
|  | (elder$ or geriatric$).ab,ti. | | |  |
|  | elder*.mp. | | |  |
|  | frail elderly.mp. or exp frail elderly/ | | |  |
|  | aged.mp. or exp Aged/ | | |  |
|  | old*.mp. | | |  |
|  | old* adult*.mp. | | |  |
|  | old* people*.mp. | | |  |
|  | >65.mp. | | |  |
|  | over 65.mp. | | |  |
|  | or/1-12 | | |  |
| **Condition** | | | | |
|  | Diabetes Mellitus, Type 2.mp. or exp Diabetes Mellitus, Type 2/ | | |  |
|  | (MODY or NIDDM or T2DM).tw,ot. | | |  |
|  | (non insulin$ depend$ or noninsulin$ depend$ or noninsulin?depend$ or noninsulin?depend).tw,ot. | | |  |
|  | ((typ$ 2 or typ$ II) adj3 diabet$).tw,ot. | | |  |
|  | ((keto?resist$ or non?keto$) adj6 diabet$).tw,ot. | | |  |
|  | (((late or adult$ or matur$ or slow or stabl$) adj3 onset) and diabet$).ab,ti. | | |  |
|  | or/14-19 | | |  |
|  | Diabetes Insipidus.mp. or exp Diabetes Insipidus/ | | |  |
|  | diabet$ insipidus.tw,ot. | | |  |
|  | 21 or 22 | | |  |
|  | 20 not 23 | | |  |
| **Intervention** | | | | |
|  | Dipeptidyl Peptidase IV Inhibitors.mp. or exp Dipeptidyl Peptidase IV Inhibitors/ | | |  |
|  | Dipeptidyl Peptidase 4.mp. or exp Dipeptidyl Peptidase 4/ | | |  |
|  | Inhibitors, Dipeptidyl-Peptidase IV.mp. | | |  |
|  | Dipeptidyl-Peptidase 4 Inhibitors.mp. | | |  |
|  | Dipeptidyl Peptidase 4 Inhibitors.mp. | | |  |
|  | Gliptins.mp. | | |  |
|  | Sitagliptin.mp. or exp Sitagliptin/ | | |  |
|  | Vildagliptin.mp. or exp Vildagliptin/ | | |  |
|  | Saxagliptin.mp. or exp Saxagliptin/ | | |  |
|  | Alogliptin.mp. or exp Alogliptin/ | | |  |
|  | Linagliptin.mp. or exp Linagliptin/ | | |  |
|  | or/25-35 | | |  |
| **Outcome** | | | | |
|  | mortality.mp. or exp mortality/ | | |  |
|  | quality of life.mp. or exp quality of life/ | | |  |
|  | QOL.mp. | | |  |
|  | cardiovascular event.mp. | | |  |
|  | myocardial infarction.mp. | | |  |
|  | stroke.mp. | | |  |
|  | hospitalization.mp. or exp hospitalization/ | | |  |
|  | hospitalisation.mp. or exp hospitalisation/ | | |  |
|  | life expectancy.mp. | | |  |
|  | cognitive impairment.mp. | | |  |
|  | cognitive status.mp. | | |  |
|  | functional status.mp. | | |  |
|  | functional impairment.mp. | | |  |
|  | renal failure.mp. | | |  |
|  | renal insufficiency.mp. or exp renal insufficiency/ | | |  |
|  | adverse drug event.mp. | | |  |
|  | adverse effects.mp. or exp adverse effects/ | | |  |
|  | drug toxicity.mp. or exp drug toxicity/ | | |  |
|  | safety.mp. | | |  |
|  | patient safety.mp. or exp patient safety/ | | |  |
|  | falls.mp. | | |  |
|  | delirium.mp. or exp delirium/ | | |  |
|  | or/37-58 | | |  |
| **Limits, Study designs** | | | | |
|  | (systematic review.ti. or meta-analysis.pt. or meta-analysis.ti. or systematic literature review.ti. or (systematic review.ti,ab. and review.pt.) or consensus development conference.pt. or practice guideline.pt. or cochrane database of systematic reviews.jn. or acp journal club.jn. or health technology assessment winchester england.jn. or evidence report technology assessment summary.jn. or drug class reviews.ti. or (clinical guideline and management).tw. or ((evidence based.ti. or evidence-based medicine.sh. or best practice*.ti. or evidence synthesis.ti,ab.) and (((review.pt. or diseases category.mp. or behaviour.sh.) and behavior mechanisms.mp.) or therapeutics.sh. or evaluation studies.pt. or validation studies.pt. or guideline.pt. or pmcbook.mp.)) or (((systematic or systematically).tw. or critical.ti,ab. or study selection.tw. or ((predetermined or inclusion) and criteri*).tw. or exclusion criteri*.tw. or main outcome measures.tw. or standard of care.tw. or standards of care.tw.) and ((survey or surveys).ti,ab. or overview*.tw. or review.ti,ab. or reviews.ti,ab. or search*.tw. or handsearch.tw. or analysis.ti,ab. or critique.ti,ab. or appraisal.tw. or (reduction.tw. and (risk.sh. or risk.tw.) and (death or recurrence).mp.)) and ((literature or articles or publications or publication or bibliography or bibliographies or published).ti,ab. or unpublished.tw. or citation.tw. or citations.tw. or database.ti,ab. or internet.ti,ab. or textbooks.ti,ab. or references.tw. or scales.tw. or papers.tw. or datasets.tw. or trials.ti,ab. or meta-analy*.tw. or (clinical and studies).ti,ab. or treatment outcome.sh. or treatment outcome.tw. or pmcbook.mp.))) not (letter or newspaper article or comment).pt. | | |  |
| **Population AND Condition AND Intervention AND Outcome AND Limits, Study designs** | | | | |
|  | 13 and 24 and 36 and 59 and 60 | | |  |

**Dipeptidyl Peptidase 4 Inhibitors in the management of Type 2 Diabetes**

**Search 2**

**Databases**

**Ovid MEDLINE(R) 1946**

**Ovid MEDLINE(R) In-Process & Other Non-Indexed Citations**

**Embase 1974**

**EBM Reviews - Health Technology Assessment**

**International Pharmaceutical Abstracts 1970**

|  | |  |  | |
| --- | --- | --- | --- | --- |
| **Population** | | | | |
|  | geriatrics.mp. or exp geriatrics/ | | |  |
|  | geriatric patient.mp. | | |  |
|  | geriatric*.mp. | | |  |
|  | (elder$ or geriatric$).ab,ti. | | |  |
|  | elder*.mp. | | |  |
|  | frail elderly.mp. or exp frail elderly/ | | |  |
|  | aged.mp. or exp Aged/ | | |  |
|  | old*.mp. | | |  |
|  | old* adult*.mp. | | |  |
|  | old* people*.mp. | | |  |
|  | >65.mp. | | |  |
|  | over 65.mp. | | |  |
|  | or/1-12 | | |  |
| **Condition** | | | | |
|  | Diabetes Mellitus, Type 2.mp. or exp Diabetes Mellitus, Type 2/ | | |  |
|  | (MODY or NIDDM or T2DM).tw,ot. | | |  |
|  | (non insulin$ depend$ or noninsulin$ depend$ or noninsulin?depend$ or noninsulin?depend).tw,ot. | | |  |
|  | ((typ$ 2 or typ$ II) adj3 diabet$).tw,ot. | | |  |
|  | ((keto?resist$ or non?keto$) adj6 diabet$).tw,ot. | | |  |
|  | (((late or adult$ or matur$ or slow or stabl$) adj3 onset) and diabet$).ab,ti. | | |  |
|  | or/14-19 | | |  |
|  | Diabetes Insipidus.mp. or exp Diabetes Insipidus/ | | |  |
|  | diabet$ insipidus.tw,ot. | | |  |
|  | 21 or 22 | | |  |
|  | 20 not 23 | | |  |
| **Intervention** | | | | |
|  | Dipeptidyl Peptidase IV Inhibitors.mp. or exp Dipeptidyl Peptidase IV Inhibitors/ | | |  |
|  | Dipeptidyl Peptidase 4.mp. or exp Dipeptidyl Peptidase 4/ | | |  |
|  | Inhibitors, Dipeptidyl-Peptidase IV.mp. | | |  |
|  | Dipeptidyl-Peptidase 4 Inhibitors.mp. | | |  |
|  | Dipeptidyl Peptidase 4 Inhibitors.mp. | | |  |
|  | Gliptins.mp. | | |  |
|  | Sitagliptin.mp. or exp Sitagliptin/ | | |  |
|  | Vildagliptin.mp. or exp Vildagliptin/ | | |  |
|  | Saxagliptin.mp. or exp Saxagliptin/ | | |  |
|  | Alogliptin.mp. or exp Alogliptin/ | | |  |
|  | Linagliptin.mp. or exp Linagliptin/ | | |  |
|  | or/25-35 | | |  |
| **Outcome** | | | | |
|  | mortality.mp. or exp mortality/ | | |  |
|  | quality of life.mp. or exp quality of life/ | | |  |
|  | QOL.mp. | | |  |
|  | cardiovascular event.mp. | | |  |
|  | myocardial infarction.mp. | | |  |
|  | stroke.mp. | | |  |
|  | hospitalization.mp. or exp hospitalization/ | | |  |
|  | hospitalisation.mp. or exp hospitalisation/ | | |  |
|  | life expectancy.mp. | | |  |
|  | cognitive impairment.mp. | | |  |
|  | cognitive status.mp. | | |  |
|  | functional status.mp. | | |  |
|  | functional impairment.mp. | | |  |
|  | renal failure.mp. | | |  |
|  | renal insufficiency.mp. or exp renal insufficiency/ | | |  |
|  | adverse drug event.mp. | | |  |
|  | adverse effects.mp. or exp adverse effects/ | | |  |
|  | drug toxicity.mp. or exp drug toxicity/ | | |  |
|  | safety.mp. | | |  |
|  | patient safety.mp. or exp patient safety/ | | |  |
|  | falls.mp. | | |  |
|  | delirium.mp. or exp delirium/ | | |  |
|  | or/37-58 | | |  |
| **Limits, Study designs** | | | | |
|  | (systematic review.ti. or meta-analysis.pt. or meta-analysis.ti. or systematic literature review.ti. or (systematic review.ti,ab. and review.pt.) or consensus development conference.pt. or practice guideline.pt. or cochrane database of systematic reviews.jn. or acp journal club.jn. or health technology assessment winchester england.jn. or evidence report technology assessment summary.jn. or drug class reviews.ti. or (clinical guideline and management).tw. or ((evidence based.ti. or evidence-based medicine.sh. or best practice*.ti. or evidence synthesis.ti,ab.) and (((review.pt. or diseases category.mp. or behaviour.sh.) and behavior mechanisms.mp.) or therapeutics.sh. or evaluation studies.pt. or validation studies.pt. or guideline.pt. or pmcbook.mp.)) or (((systematic or systematically).tw. or critical.ti,ab. or study selection.tw. or ((predetermined or inclusion) and criteri*).tw. or exclusion criteri*.tw. or main outcome measures.tw. or standard of care.tw. or standards of care.tw.) and ((survey or surveys).ti,ab. or overview*.tw. or review.ti,ab. or reviews.ti,ab. or search*.tw. or handsearch.tw. or analysis.ti,ab. or critique.ti,ab. or appraisal.tw. or (reduction.tw. and (risk.sh. or risk.tw.) and (death or recurrence).mp.)) and ((literature or articles or publications or publication or bibliography or bibliographies or published).ti,ab. or unpublished.tw. or citation.tw. or citations.tw. or database.ti,ab. or internet.ti,ab. or textbooks.ti,ab. or references.tw. or scales.tw. or papers.tw. or datasets.tw. or trials.ti,ab. or meta-analy*.tw. or (clinical and studies).ti,ab. or treatment outcome.sh. or treatment outcome.tw. or pmcbook.mp.))) not (letter or newspaper article or comment).pt. | | |  |
| **Population AND Condition AND Intervention AND Outcome AND Limits, Study designs** | | | | |
|  | 13 and 24 and 36 and 59 and 60 | | |  |

**Dipeptidyl Peptidase 4 Inhibitors in the management of Type 2 Diabetes**

**Search 3B (**[**Cochrane handbook**](http://handbook.cochrane.org/) **and** [**Fraser 2006**](http://www.ncbi.nlm.nih.gov/pubmed/16919159) **for MEDLINE)**

**Databases**

**Ovid MEDLINE(R) 1946**

**Ovid MEDLINE(R) In-Process & Other Non-Indexed Citations**

**EBM Reviews - Health Technology Assessment**

**International Pharmaceutical Abstracts**

|  | |  |  | |
| --- | --- | --- | --- | --- |
| **Population** | | | | |
|  | geriatrics.mp. or exp geriatrics/ | | |  |
|  | geriatric patient.mp. | | |  |
|  | geriatric*.mp. | | |  |
|  | (elder$ or geriatric$).ab,ti. | | |  |
|  | elder*.mp. | | |  |
|  | frail elderly.mp. or exp frail elderly/ | | |  |
|  | aged.mp. or exp Aged/ | | |  |
|  | old*.mp. | | |  |
|  | old* adult*.mp. | | |  |
|  | old* people*.mp. | | |  |
|  | >65.mp. | | |  |
|  | over 65.mp. | | |  |
|  | or/1-12 | | |  |
| **Condition** | | | | |
|  | Diabetes Mellitus, Type 2.mp. or exp Diabetes Mellitus, Type 2/ | | |  |
|  | (MODY or NIDDM or T2DM).tw,ot. | | |  |
|  | (non insulin$ depend$ or noninsulin$ depend$ or noninsulin?depend$ or noninsulin?depend).tw,ot. | | |  |
|  | ((typ$ 2 or typ$ II) adj3 diabet$).tw,ot. | | |  |
|  | ((keto?resist$ or non?keto$) adj6 diabet$).tw,ot. | | |  |
|  | (((late or adult$ or matur$ or slow or stabl$) adj3 onset) and diabet$).ab,ti. | | |  |
|  | or/14-19 | | |  |
|  | Diabetes Insipidus.mp. or exp Diabetes Insipidus/ | | |  |
|  | diabet$ insipidus.tw,ot. | | |  |
|  | 21 or 22 | | |  |
|  | 20 not 23 | | |  |
| **Intervention** | | | | |
|  | Dipeptidyl Peptidase IV Inhibitors.mp. or exp Dipeptidyl Peptidase IV Inhibitors/ | | |  |
|  | Dipeptidyl Peptidase 4.mp. or exp Dipeptidyl Peptidase 4/ | | |  |
|  | Inhibitors, Dipeptidyl-Peptidase IV.mp. | | |  |
|  | Dipeptidyl-Peptidase 4 Inhibitors.mp. | | |  |
|  | Dipeptidyl Peptidase 4 Inhibitors.mp. | | |  |
|  | Gliptins.mp. | | |  |
|  | Sitagliptin.mp. or exp Sitagliptin/ | | |  |
|  | Vildagliptin.mp. or exp Vildagliptin/ | | |  |
|  | Saxagliptin.mp. or exp Saxagliptin/ | | |  |
|  | Alogliptin.mp. or exp Alogliptin/ | | |  |
|  | Linagliptin.mp. or exp Linagliptin/ | | |  |
|  | or/25-35 | | |  |
| **Outcome** | | | | |
|  | mortality.mp. or exp mortality/ | | |  |
|  | quality of life.mp. or exp quality of life/ | | |  |
|  | QOL.mp. | | |  |
|  | cardiovascular event.mp. | | |  |
|  | myocardial infarction.mp. | | |  |
|  | stroke.mp. | | |  |
|  | hospitalization.mp. or exp hospitalization/ | | |  |
|  | hospitalisation.mp. or exp hospitalisation/ | | |  |
|  | life expectancy.mp. | | |  |
|  | cognitive impairment.mp. | | |  |
|  | cognitive status.mp. | | |  |
|  | functional status.mp. | | |  |
|  | functional impairment.mp. | | |  |
|  | renal failure.mp. | | |  |
|  | renal insufficiency.mp. or exp renal insufficiency/ | | |  |
|  | adverse drug event.mp. | | |  |
|  | adverse effects.mp. or exp adverse effects/ | | |  |
|  | drug toxicity.mp. or exp drug toxicity/ | | |  |
|  | safety.mp. | | |  |
|  | patient safety.mp. or exp patient safety/ | | |  |
|  | falls.mp. | | |  |
|  | delirium.mp. or exp delirium/ | | |  |
|  | or/37-58 | | |  |
| **Limits, Study designs** | | | | |
|  | | randomized controlled trial.pt. |  | |
|  | | controlled clinical trial.pt. |  | |
|  | | randomized.ab. |  | |
|  | | placebo.ab. |  | |
|  | | drug therapy.fs. |  | |
|  | | randomly.ab. |  | |
|  | | trial.ab. |  | |
|  | | groups.ab. |  | |
|  | | or/60-67 |  | |
|  | | exp animals/ not humans.sh. |  | |
|  | | 68 not 69 |  | |
|  | | Comparative studies/ |  | |
|  | | Follow-up studies/ |  | |
|  | | Time factors/ |  | |
|  | | chang$.tw. |  | |
|  | | evaluat$.tw. |  | |
|  | | reviewed.tw. |  | |
|  | | prospective$.tw. |  | |
|  | | retrospective$.tw. |  | |
|  | | baseline.tw. |  | |
|  | | cohort.tw. |  | |
|  | | case series.tw. |  | |
|  | | or/70-81 |  | |
| **Population AND Condition AND Intervention AND Outcome AND Limits, Study designs** | | | | |
|  | 13 and 24 and 36 and 59 and 82 | | |  |

**Dipeptidyl Peptidase 4 Inhibitors in the management of Type 2 Diabetes**

**Search 3B (**[**Cochrane handbook**](http://handbook.cochrane.org/) **and** [**Fraser 2006**](http://www.ncbi.nlm.nih.gov/pubmed/16919159) **for EMBASE)**

**Database**

**Embase 1974**

|  | |  |  | |
| --- | --- | --- | --- | --- |
| **Population** | | | | |
|  | geriatrics.mp. or exp geriatrics/ | | |  |
|  | geriatric patient.mp. | | |  |
|  | geriatric*.mp. | | |  |
|  | (elder$ or geriatric$).ab,ti. | | |  |
|  | elder*.mp. | | |  |
|  | frail elderly.mp. or exp frail elderly/ | | |  |
|  | aged.mp. or exp Aged/ | | |  |
|  | old*.mp. | | |  |
|  | old* adult*.mp. | | |  |
|  | old* people*.mp. | | |  |
|  | >65.mp. | | |  |
|  | over 65.mp. | | |  |
|  | or/1-12 | | |  |
| **Condition** | | | | |
|  | Diabetes Mellitus, Type 2.mp. or exp Diabetes Mellitus, Type 2/ | | |  |
|  | (MODY or NIDDM or T2DM).tw,ot. | | |  |
|  | (non insulin$ depend$ or noninsulin$ depend$ or noninsulin?depend$ or noninsulin?depend).tw,ot. | | |  |
|  | ((typ$ 2 or typ$ II) adj3 diabet$).tw,ot. | | |  |
|  | ((keto?resist$ or non?keto$) adj6 diabet$).tw,ot. | | |  |
|  | (((late or adult$ or matur$ or slow or stabl$) adj3 onset) and diabet$).ab,ti. | | |  |
|  | or/14-19 | | |  |
|  | Diabetes Insipidus.mp. or exp Diabetes Insipidus/ | | |  |
|  | diabet$ insipidus.tw,ot. | | |  |
|  | 21 or 22 | | |  |
|  | 20 not 23 | | |  |
| **Intervention** | | | | |
|  | Dipeptidyl Peptidase IV Inhibitors.mp. or exp Dipeptidyl Peptidase IV Inhibitors/ | | |  |
|  | Dipeptidyl Peptidase 4.mp. or exp Dipeptidyl Peptidase 4/ | | |  |
|  | Inhibitors, Dipeptidyl-Peptidase IV.mp. | | |  |
|  | Dipeptidyl-Peptidase 4 Inhibitors.mp. | | |  |
|  | Dipeptidyl Peptidase 4 Inhibitors.mp. | | |  |
|  | Gliptins.mp. | | |  |
|  | Sitagliptin.mp. or exp Sitagliptin/ | | |  |
|  | Vildagliptin.mp. or exp Vildagliptin/ | | |  |
|  | Saxagliptin.mp. or exp Saxagliptin/ | | |  |
|  | Alogliptin.mp. or exp Alogliptin/ | | |  |
|  | Linagliptin.mp. or exp Linagliptin/ | | |  |
|  | or/25-35 | | |  |
| **Outcome** | | | | |
|  | mortality.mp. or exp mortality/ | | |  |
|  | quality of life.mp. or exp quality of life/ | | |  |
|  | QOL.mp. | | |  |
|  | cardiovascular event.mp. | | |  |
|  | myocardial infarction.mp. | | |  |
|  | stroke.mp. | | |  |
|  | hospitalization.mp. or exp hospitalization/ | | |  |
|  | hospitalisation.mp. or exp hospitalisation/ | | |  |
|  | life expectancy.mp. | | |  |
|  | cognitive impairment.mp. | | |  |
|  | cognitive status.mp. | | |  |
|  | functional status.mp. | | |  |
|  | functional impairment.mp. | | |  |
|  | renal failure.mp. | | |  |
|  | renal insufficiency.mp. or exp renal insufficiency/ | | |  |
|  | adverse drug event.mp. | | |  |
|  | adverse effects.mp. or exp adverse effects/ | | |  |
|  | drug toxicity.mp. or exp drug toxicity/ | | |  |
|  | safety.mp. | | |  |
|  | patient safety.mp. or exp patient safety/ | | |  |
|  | falls.mp. | | |  |
|  | delirium.mp. or exp delirium/ | | |  |
|  | or/37-58 | | |  |
| **Limits, Study designs** | | | | |
|  | | random$.mp. |  | |
|  | | factorial$.mp. |  | |
|  | | crossover$.mp. |  | |
|  | | cross over$.mp. |  | |
|  | | cross-over$.mp. |  | |
|  | | placebo$.mp. |  | |
|  | | (doubl$ adj blind$).mp. |  | |
|  | | (singl$ adj blind$).mp. |  | |
|  | | assign$.mp. |  | |
|  | | allocat$.mp. |  | |
|  | | volunteer$.mp. |  | |
|  | | crossover procedure/ |  | |
|  | | double blind procedure/ |  | |
|  | | randomized controlled trial/ |  | |
|  | | single blind procedure/ |  | |
|  | | or/60-74 |  | |
|  | | Controlled study/ |  | |
|  | | Treatment outcome/ |  | |
|  | | Major clinical study/ |  | |
|  | | Clinical trial/ |  | |
|  | | chang$.tw. |  | |
|  | | evaluat$.tw. |  | |
|  | | reviewed.tw. |  | |
|  | | baseline.tw. |  | |
|  | | (compare$ or compara$).tw. |  | |
|  | | or/75-84 |  | |
| **Population AND Condition AND Intervention AND Outcome AND Limits, Study designs** | | | | |
|  | 13 and 24 and 36 and 59 and 85 | | |  |
