# Supplementary material for: Effectiveness and safety of dipeptidyl peptidase 4 inhibitors in the management of type 2 diabetes in older adults: a systematic review and development of recommendations to reduce inappropriate prescribing
Source: BMC Geriatr. 2017 Oct 16;17(Suppl 1):226. doi: 10.1186/s12877-017-0571-8 (PMC5647559; doi:10.1186/s12877-017-0571-8)
Supplement: Supplementary file 3 — Participant characteristics. Characteristics of the participants in the included studies. (DOCX 60 kb) [file 12877_2017_571_MOESM3_ESM.docx]

**Table S1 - Characteristics of participants in included studies**

| **Authors and publication year** | **Setting / country / ethnicity** | **Male sex** | **Age** | **Reported comorbidities** | **Reported concomitant medications** | **Functional status / Frailty level** | **Cognitive status** |
| --- | --- | --- | --- | --- | --- | --- | --- |
| Johansen et al. 2012 | P using linagliptin:  White: 59.7%  Black: 1.4%  Asian: 38.9%  P using comparators:  White: 61.8%  Black: 1.6%  Asian: 36.6% | P using linagliptin: 53.7%  P using comparators: 58.6% | P using linagliptin: 58 (10)^a^  P using comparators: 58 (10) ^a^ | P using linagliptin:  Metabolic syndrome: 60.3%  Coronary artery disease: 10.4%  Cerebrovascular disease: 2.9%  Peripheral artery disease: 2.3%  Hypertension: 63.8%  P using comparators:  Metabolic syndrome: 61.7%  Coronary artery disease: 11.0%  Cerebrovascular disease: 3.9%  Peripheral artery disease: 3.0%  Hypertension: 66.0% | P using linagliptin:  ASA: 29.5%  Antihypertensive: 60.0%  Lipid-lowering therapy: 39.5%  Any of the above: 72.8%  P using comparators:  ASA: 30.5%  Antihypertensive: 63.0%  Lipid-lowering therapy: 42.1%  Any of the above: 75.5% | Not reported | Not reported |
| Banerji et al. 2010 | Primary care USA  P with normal renal function:  Caucasian: ~53%  Non-Caucasian: ~48%  Black: ~16%  Hispanic/Latino: ~26%  Other: ~12%  P with mild impaired renal function:  Caucasian: ~72%  Non-Caucasian: ~29%  Black: ~10%  Hispanic/Latino: ~14%  Other: ~18% | P with normal renal function:  Vilda + Met: 51.5%  TZD + Met: 52.7%  P with mild impaired renal function:  Vilda + Met: 51.9%  TZD + Met: 51.5% | P with normal renal function:  Vilda + Met: 53.1 (10.23)^a^  TZD + Met: 54.3 (10.23) ^a^  P with mild impaired renal function:  Vilda + Met: 61.3 (8.5) ^a^  TZD + Met: 61.1 (8.3) ^a^ | Not reported | Not reported | Not reported | Not reported |
| Barnett et al. 2013 | Five countries: Australia, Canada, Denmark, the Netherlands, Sweden  P using linagliptin:  White: 96.9%  Asian: 1.9%  Black: 1.2%  P using placebo:  White: 96.2%  Asian: 2.5%  Black: 1.3% | P using linagliptin: 71.6%  P using placebo: 62.0% | P using linagliptin: 74.9 (4.4)^a^  P using placebo: 74.9 (4.2) ^a^ | Mean Charlson age-comorbiditiy score 5.1 (0.7)^a^ | >5 drugs (diabetes and non-diabetes):  P using linagliptin: 67.9%  P using placebo: 77.2% | Not reported | Not reported |
| Barzilai et al. 2011 | USA  P using sitagliptin:  White: 83%  Black: 9%  Hispanic: 6%  Asian: 3%  Other: 0  P using placebo:  White: 75%  Black: 10%  Hispanic: 9%  Asian: 3%  Other: 4% | P using sitagliptin: 47%  P using placebo: 47% | P using sitagliptin: 71.6 (6.1)^a^  P using placebo: 72.1 (6.0) ^a^ | All randomised patients had at least one secondary diagnosis; the most common of these were hypertension, hyperlipidaemia, osteoarthritis, and gastroesophageal reflux. | Not reported | Not reported | Not reported |
| Chien et al. 2011 | Taiwan | P using sitagliptin: 36.7%  P using OAD combinations: 47.9% | P using sitagliptin: 73.5 (5.9) ^a^  P using OAD combinations: 72.5 (5.2) ^a^ | Not reported | Not reported | Not reported | Not reported |
| Ferrannini et al. 2009 | P using vildagliptin:  Caucasian: 86.3%  Black: 1.3%  Asian: 3.2%  Hispanic/Latino: 8.9%  Others: 0.4%  P using glimepiride:  Caucasian: 85.2%  Black: 1.4%  Asian: 3.2%  Hispanic/Latino: 9.3%  Others: 1.0% | P using vildagliptin: 52.8%  P using glimepiride: 54.1% | P using vildagliptin: 57.50 (9.06) ^a^  P using glimepiride: 57.46 (9.28) ^a^ | P using vildagliptin:  Mild renal insufficiency: 44.7%  Moderate renal insufficiency: 4.7%  Hypertension, 64.6%  Dyslipidaemia: 49.3%  Previous cardiac disorder: 19.2%  P using glimepiride:  Mild renal insufficiency: 43.1%  Moderate renal insufficiency: 5.0%  Hypertension: 68.5%  Dyslipidaemia: 50.0%  Previous cardiac disorder: 19.6% | Concomitant medications:  P using vildagliptin: 93.1%  P using glimepiride: 93.9%  In both groups:  Antihypertensive agents: ACE-I: ~ 43%  ARB and beta-blockers (alone or in combination with diuretics): 22–24%  Lipid-lowering agents: 47% (mostly statins: ~42%)  Platelet aggregation inhibitors: third of patients | Not reported | Not reported |
| Fonseca et al. 2008 | P using vildagliptin 100 mg/d + insulin:  Caucasian: 72.9%  Black: 14.6%  Hispanic/Latino: 10.4%  Asian: 2.1%  P using placebo + insulin / vildagliptin 50 mg/d + insulin:  Caucasian: 69.2%  Black: 11.5%  Hispanic/Latino: 17.3%  Asian: 1.9% | P using vildagliptin 100 mg/d + insulin: 50%  P using placebo + insulin / vildagliptin 50 mg/d + insulin: 52.9% | P using vildagliptin 100 mg/d + insulin: 59.95 (9.58) ^a^  P using placebo + insulin / vildagliptin 50 mg/d + insulin: 58.19 (10.84) ^a^ | In both groups:  Hypertension: more than two-thirds  Diabetic complications: particularly neuropathy  Dyslipidaemia: no data reported | Not reported | Not reported | Not reported |
| Green et al 2015 | 38 countries.  P using sitagliptin:  White: 67.6%  Black: 2.8%  Asian: 22.6%  Other: 7.1%  Hispanic or Latino: 12.1%  P using placebo:  White: 68.2%  Black: 3.3%  Asian: 22.0%  Other: 6.6%  Hispanic or Latino: 12.4% | P using sitagliptin: 70.9%  P using placebo: 70.5% | P using sitagliptin: 65.4 (7.9) ^a^  P using placebo: 65.5 (8.0) ^a^ | P using sitagliptin:  Prior cardiovascular disease: 73.6%  Prior cerebrovascular disease: 24.6%  Prior peripheral arterial disease: 16.6%  Prior congestive heart failure: 17.8%  P using placebo:  Prior cardiovascular disease: 74.5%  Prior cerebrovascular disease: 24.3%  Prior peripheral arterial disease: 16.6%  Prior congestive heart failure: 18.3% | P using sitagliptin:  Metformin: 81.0%  SU: 45.6%  TZD: 2.7%  Insulin: 23.5%  Beta blocker: 63.4% ACE-Ior ARB: 78.3%  CCB: 33.3%  Diuretic: 40.6%  Aspirin: 78.6%  Other antiplatelet: 21.7% Statin: 79.8%  Ezetimibe: 5.3% | Not reported | Not reported |
| Hartley et al. 2015 | 85 sites in 13 countries  Sitagliptin:  White: 61.4%  Multi-racial: 24.4%  Native American/Alaska Native: 9.1%  Asian: 2.5%  African American: 2.0%  Native Hawaiian/Pacific Islander: 0.5%  Glimepiride:  White: 53.9%  Multi-racial: 31.9%  Native American/Alaska Native: 7.9%  Asian: 6.3%  African American: 0.0%  Native Hawaiian/Pacific Islander: 0.0% | Sitagliptin:  47.2%  Glimepiride:  40.3% | Sitagliptin:  70.6 (4.8) ^a^  Glimepiride:  70.8 (4.9) ^a^ | Not reported | Not reported | Not reported | Not reported |
| Kadowaki et al. 2014 | Japan | P using teneligliptin + glimepiride: 64.6%  P using placebo + glimepiride: 67.3% | P using teneligliptin + glimepiride: 58.4 (8.6) ^a^  P using placebo + glimepiride: 60.3 (7.8) ^a^ | Not reported | Not reported | Not reported | Not reported |
| Matthews et al. 2010 | P using vildagliptin:  Caucasian: 87.3%  Black: 1.2%  Asian: 2.8%  Hispanic/Latino: 8.3%  Other: 0.4%  P using glimepiride:  Caucasian: 86.3%  Black: 1.2%  Asian: 3.0%  Hispanic/Latino: 8.5%  Other: 1.0% | P using vildagliptin: 53.1%  P using glimepiride: 53.9%) | P using vildagliptin: 57.5 (9.07) ^a^  P using glimepiride: 57.5 (9.19) ^a^ | Not reported | In both groups: 96% treated with other medications in addition to study drugs  Antihypertensive agents: ACE-I: 46%  ARB (alone or in combination with diuretics): 26%  Beta-blockers: 25%  Lipid lowering agents: 46% (mostly statins)  Platelet aggregation inhibitors: 36% | Not reported | Not reported |
| Rosenstock et al. 2013 | P using alogliptin:  American Indian or Alaska Native: 5.4%  Asian: 8.6%  Black or African  American: 7.2%  White: 76.1%  70.3%  Multiracial: 2.7%  P using glipizide:  American Indian or Alaska Native:  5.9%  Asian:  11.9%  Black or African  American: 9.1%  White: 70.3%  Multiracial: 2.7% | P using alogliptin: 45.9%  P using glipizide: 43.8% | P using alogliptin: 70.1 (4.42) ^a^  P using glipizide: 69.8 (4.07) ^a^ | In both groups:  Hypertension: 68%  Hyperlipidaemia: 21%  Osteoarthritis: 20%  Dyslipidaemia: 15%  Obesity: 11% | In both groups:  Agents acting on the renin-angiotensin system: 54%  Lipid modifying agents: 47%  Aspirin: 41% | Not reported | Not reported |
| Schernthaner et al. 2015 | 152 sites in 12 European countries and Mexico.  P using saxagliptin + metformin: Central Europe: 36.9%  Latin countries: 20.6%  Nordic countries: 42.5%  White: 97.8%  Other: 2.2%  P using glimepiride + metformin:  Central Europe: 39.2%  Latin countries: 17.2%  Nordic countries: 43.6%  White: 98.6%  Other: 1.4% | P using saxagliptin + metformin: 60.3%  P using glimepiride + metformin: 63.3% | P using saxagliptin + metformin: 72.5 (5.7) ^a^  P using glimepiride + metformin: 72.7 (5.4) ^a^ | P using saxagliptin + metformin:  Hypertension: 76.7%  Coronary artery disease: 8.6%  Previous MI: 9.4%  CV accident: 5.3%  Stable angina: 4.7%  History of lipid disorder: 61.1  P using glimepiride + metformin:  Hypertension: 77.5%  Coronary artery disease: 10.0%  Previous MI: 5.6%  CV accident: 5.8%  Stable angina 5.8%  History of lipid disorder: 59.2% | Not reported | Not reported | Not reported |
| Schweizer et al. 2009 | 14 countries in Europe, the Americas and Asia.  P using vildagliptin:  Caucasian: 72.8%  Asian: 18.9%  Hispanics: 7.7%  All others: 0.6%  P using metformin: Caucasian: 70.5%  Asian: 21.7%  Hispanics: 6.0%  All others: 1.8% | P using vildagliptin: 44.4%  P using metformin: 53.0% | P using vildagliptin:  71.6 (5.2) ^a^  P using metformin: 70.2 (5.1) ^a^ | Overall:  Hypertension: >70%  Dyslipidaemia: 40%  Mild renal insufficiency: >50%  Cardiac disorders: 33% | Overall: 92% received concomitant medications  ACE-I (mostly combined with diuretics): ~37%  Beta-blockers: 28%  CCB: 20%  ARB (alone or in combination with diuretics): 19%  Lipid-lowering agents: 39% (mostly statins)  Platelet aggregation inhibitors: third of patients | Not reported | Not reported |
| Schweizer et al. 2013 | P using vildagliptin:  White: 88.0%  Asian: 6.0%  Hispanic/Latino: 6.0%  All other: 0  P using metformin:  White: 78.2%  Asian: 9.1%  Hispanic/Latino: 10.9%  All other: 1.8% | P using vildagliptin: 52.0%  P using metformin: 54.5% | P using vildagliptin: 78.0 (2.5) ^a^  P using metformin: 78.3 (2.7) ^a^ | P using vildagliptin:  Hypertension: 96.0%  Dyslipidaemia: 72.0%  High CV risk status: 52.0%  P using metformin: Hypertension: 96.4%  Dyslipidaemia: 65.5%  High CV risk status: 54.5% | P using vildagliptin:  ≥5 medications: 84.0%  Anti-hypertensive medications: 94.0%  Lipid-lowering medications: 68.0%  P using metformin:  ≥5 medications: 85.5%  Anti-hypertensive medications: 96.4%  Lipid-lowering medications: 61.8% | Not reported | Not reported |
| Scirica et al. 2013  Scirica 2014 et al.  Leiter et al. 2015  Mosenzon et al. 2015 | 788 sites in 26 countries.  P using saxagliptin:  White: 75.4%  Hispanic: 21.5%  P using placebo:  White: 75.1%  Hispanic: 21.5% | P using saxagliptin: 66.6%  P using placebo: 67.2% | P using saxagliptin: 65.1 (8.5)^a^  P using placebo: 65.0 (8.6) ^a^ | P using saxagliptin:  Established atherosclerotic disease: 78.4%  Hypertension: 81.2%  Dyslipidaemia: 71.2%  Prior MI: 38.0%  Prior HF: 12.8%  Prior coronary revascularization: 43.1%  P using placebo:  Established atherosclerotic disease: 78.7%  Hypertension: 82.4%  Dyslipidaemia: 71.2%  Prior MI: 37.6%  Prior HF: 12.8%  Prior coronary revascularization: 43.3% | P using saxagliptin:  Aspirin: 75.5%  Statin: 78.3%  ACE-I: 53.6%  ARB: 28.2%  Beta-blockers: 61.6%  Metformin: 69.9%  SU: 40.5%  TZD: 6.2%  Insulin: 41.6%  OAM: 0.6%  NAM: 4.1%  P using placebo:  Aspirin: 75.0%  Statin: 78.4%  ACE-I: 54.9%  ARB: 27.6%  Beta-blockers: 61.6%  Metformin: 69.2%  SU: 40.0%  TZD: 5.7%  Insulin: 41.2%  OAM: 0.6%  NAM: 4.8% | Not reported | Not reported |
| Strain et al. 2013 | P using vildagliptin:  White: 97.1%  Other: 2.9%  P using placebo:  White: 96.4%  Other: 3.6% | P using vildagliptin: 52.5 %  P using placebo: 38.1% | P using vildagliptin: 75.1 (4.3) ^a^  P using placebo: 74.4 (4.0) ^a^ | P using vildagliptin: Hypertension: 84·2%  Dyslipidaemia: 26·6%  Hypercholesterolemia: 15·8%  Hyper lipidaemia: 25·9%  Myocardial ischaemia: 15·8%  Peripheral neuropathy: 21·6%  Osteoarthritis: 19·4%  P using plcaebo: Hypertension: 82·7%  Dyslipidaemia: 20·9%  Hypercholesterolemia: 15·8%  Hyper lipidaemia: 20·1%  Myocardial ischaemia: 17·3%  Peripheral neuropathy: 25·9%  Osteoarthritis: 20·1% | Almost all patients were using concomitant medications, with a substantial majority taking antihypertensive and lipid-lowering medications | Frailty status  P using vildagliptin: Yes: 8.6%  No: 90.6%  P using placebo:  Yes: 10.1%  No: 88.5% | Not reported |
| White et al. 2013 | 898 centres in 49 countries  P using alogliptin:  White: 72.8%  Black: 3.7%  Asian: 20.3%  Native American: 2.1%  Other: 1.1%  P using placebo:  White: 72.5%  Black: 4.3%  Asian: 20.2%  Native American: 2.0%  Other: 0.9% | P using alogliptin: 67.7%  P using placebo: 68.0% | P using alogliptin:  61.0^b^  P using placebo: 61.0^b^ | P using alogliptin:  Hypertension: 82.5%  MI: 88.4%  Percutaneous coronary intervention: 62.5%  Coronary-artery bypass grafting: 12.8%  Congestive heart failure: 28.0%  Stroke: 7.2%  Peripheral arterial disease: 9.7%  P using placebo:  Hypertension: 83.6%  MI: 87.5%  Percutaneous coronary intervention: 62.8%  Coronary-artery bypass grafting: 12.7%  Congestive heart failure: 27.8%  Stroke: 7.2%  Peripheral arterial disease: 9.4% | P using alogliptin:  Antiplatelet agents: 97.4%  Aspirin: 90.6%  Thienopyridine: 79.8%  Beta-blockers: 81.7%  Statins: 90.6%  Antidiabetic agents: 99.1%  Insulin: 29.4%  Metformin: 65.0%  TZD: 2.5%  SU: 46.9%  CCB: 21.7%  Diuretics: 37.2%  Renin–angiotensin system-blocking agents: 81.5%  P using placebo:  Antiplatelet agents: 97.1%  Aspirin: 90.8%  Thienopyridine: 80.8%  Beta-blockers: 82.2%  Statins: 90.3%  Antidiabetic agents: 98.9%  Insulin: 30.3%  Metformin: 67.4%  TZD: 2.4%  SU: 46.2%  CCB: 22.8%  Diuretics: 37.7%  Renin–angiotensin system–blocking agents: 82.5% | Not reported | Not reported |
| Chang et al. 2015 | Taiwan | DPP-4 inhibitors + metformin: 51.0%  SU + metformin: 53.6%  Glinides + metformin: 55.3%  Pioglitazone + metformin: 51.6%  α-glucosidase inhibitor + metformin: 56.6% | DPP-4 inhibitors + metformin: 58 (17)^c^  SU + metformin: 58 (18)^c^  Glinides + metformin: 60 (20)^c^  Pioglitazone+ metformin:  58 (17)^c^  α-glucosidase inhibitor + metformin: 58 (18)^c^ | DPP-4 inhibitors + metformin:  Ischaemic heart disease: 17.1%  Cerebrovascular disease: 9.5%  CRD: 0.7%  Hypertension: 57.9% Dyslipidaemia: 56.2%  SU + metformin:  Ischaemic heart disease: 14.7%  Cerebrovascular disease: 8.6%  CRD: 1.0%  Hypertension: 59.4% Dyslipidaemia: 51.3%  Glinides + metformin:  Ischaemic heart disease: 18.3%  Cerebrovascular disease: 12.4%  CRD: 1.3 %  Hypertension: 59.2% Dyslipidaemia: 51.1%  Pioglitazone + metformin:  Ischaemic heart disease: 15.0%  Cerebrovascular disease: 8.2%  CRD: 0.9%  Hypertension: 60.7% Dyslipidaemia: 56.4%  α-glucosidase inhibitor + metformin:  Ischaemic heart disease: 18.0%  Cerebrovascular disease: 8.4%  CRD: 1.5%  Hypertension: 60.4% Dyslipidaemia: 54.1% | DPP-4 inhibitors + metformin:  Aspirin: 28.1% Clopidogrel: 4.2% Warfarin: 1.5%  α-blockers: 4.2%  ACE-I: 12.9%  ARB: 31.0%  Beta-blockers: 33.2% CCB: 38.8%  Diuretics: 16.4%  Nitrates: 10.4%  Statins: 40.5%  Fibrates: 14.4%  SU + metformin:  Aspirin: 26.7% Clopidogrel: 2.2% Warfarin: 0.9%  α-blockers: 4.0%  ACE-I: 17.5%  ARB: 20.3%  Beta-blockers: 31.8% CCB: 41.6%  Diuretics: 19.3%  Nitrates: 8.9%  Statins: 30.4%  Fibrates: 13.9%  Glinides + metformin:  Aspirin: 31.0% Clopidogrel: 3.2% Warfarin: 1.2%  α-blockers: 5.0%  ACE-I: 18.4%  ARB: 22.6%  Beta-blockers: 31.5% CCB: 42.6%  Diuretics: 21.1%  Nitrates: 11.9%  Statins: 32.0%  Fibrates: 12.5%  Pioglitazone + metformin:  Aspirin: 28.1% Clopidogrel: 2.8% Warfarin: 1.1%  α-blockers: 3.8%  ACE-I: 15.8%  ARB: 25.5%  Beta-blockers: 35.6% CCB: 40.3%  Diuretics: 19.4%  Nitrates: 10.4%  Statins: 36.3%  Fibrates: 14.9%  a-glucosidase inhibitor + metformin:  Aspirin: 26.3% Clopidogrel: 2.2% Warfarin: 0.5%  α-blockers: 3.0%  ACE-I: 17.7%  ARB: 25.7%  Beta-blockers: 34.1% CCB: 39.6%  Diuretics: 15.7%  Nitrates: 9.4%  Statins: 36.6%  Fibrates: 14.5% | Not reported | Not reported |
| Chen et al. 2015 | Taiwan | Sitagliptin: 56.3%  Comparison: 56.9% | Sitagliptin: 67.6 (11.1)^a^  Comparison: 67.5 (10.9)^a^ | Sitagliptin:  Neuropathy: 18.1%  Retinopathy: 6.1%  Coronary artery disease: 14.1%  CKD: 5.9%  COPD: 13.3%  AF: 5.9%  Peripheral arterial disease: 9.6%  Hypertension: 85.5%  HF: 6.9%  Dyslipidaemia: 56.9%  Malignancy: 5.9%  Cirrhosis: 2.4%  Comparison:  Neuropathy: 16.3%  Retinopathy: 6.1%  Coronary artery disease: 13.7%  CKD: 6.4%  COPD: 14.3%  AF: 6.1%  Peripheral arterial disease: 9.6%  Hypertension: 84.8%  HF: 7.0%  Dyslipidaemia: 56.3%  Malignancy: 5.6%  Cirrhosis: 2.0% | Sitagliptin:  Insulin: 16.5%  Metformin: 60.0%  TZD: 10.2%  SU: 62.2%  Aspirin: 74.2%  Clopidogrel: 36.6%  Warfarin: 5.5%  Beta-blockers: 26.1%  ACE-I or ARB: 65.2%  CCB: 44.3%  Diuretics: 18.7%  Statins: 41.6%  Fibrate: 8.1%  Comparison:  Insulin: 17.3%  Metformin: 59.9%  TZD: 9.4%  SU: 61.8%  Aspirin: 74.1%  Clopidogrel: 36.0%  Warfarin: 5.5%  Beta-blockers: 26.1%  ACE-I or ARB: 64.8%  CCB: 45.0%  Diuretics: 19.2%  Statins: 42.9%  Fibrate: 8.3% | Not reported | Not reported |
| Driessen et al. 2014 | Primary care  UK | P using NIAD: 52.7%  P without prescription of NIAD: 52.7%  P using DPP-4 inhibitor: 57.0% | P using NIAD: 61 (21.0)^a^  P without prescription of NIAD: 61 (21.0)^a^  P using DPP-4 inhibitor: 59 (16.0)^a^ | P using NIAD:  Fracture: 20.7%  Hyperthyroidism: 1.0%  Hypothyroidism: 7.9%  COPD: 5.5%  CHF: 4.2%  Cancer: 21.8%  Rheumatoid arthritis: 1.7%  Retinopathy: 12.1%  Secondary osteoporosis: 9.0%  Neuropathy: 7.6%  P without prescription of NIAD:  Fracture: 20.8%  Hyperthyroidism: 0.8%  Hypothyroidism: 5.2%  COPD: 4.6%  CHF: 1.9%  Cancer: 23.1%  Rheumatoid arthritis: 1.6%  Retinopathy: 0.6%  Secondary osteoporosis: 3.6%  Neuropathy: 1.3%  P using DPP-4 inhibitor:  Fracture: 20.9%  Hyperthyroidism: 0.9%  Hypothyroidism: 7.7%  COPD: 4.3%  CHF: 2.7%  Cancer: 20.7%  Rheumatoid arthritis: 1.5%  Retinopathy: 12.7%  Secondary osteoporosis: 6.5%  Neuropathy: 7.1% | P using NIAD^d^:  Metformin: 82.6%  SU derivatives: 26.8%  TZD: 10.4%  Insulins: 11.2%  Oral glucocorticoids: 5.8%  Statins: 54.7%  Antiarrhythmics: 1.6%  Antidepressants: 16.1%  Anti-Parkinson: 0.5%  Antipsychotics: 2.1%  Anxiolytics: 7.1%  Hypnotics: 4.9%  Antihypertensives: 57.8%  P without prescription of NIAD:  Metformin: N/A  SU derivatives: N/A  TZD: N/A  Insulins: N/A  Oral glucocorticoids: 4.0%  Statins: 20.9%  Antiarrhythmics: 1.3%  Antidepressants: 10.5%  Anti-Parkinson: 0.6%  Antipsychotics: 1.2%  Anxiolytics: 5.8%  Hypnotics: 4.2%  Antihypertensives: 32.7%  P using DPP-4 inhibitor:  Metformin: 88.2%  SU derivatives: 38.6%  TZD: 20.5%  Insulins: 4.4%  Oral glucocorticoids: 4.7%  Statins: 68.2%  Antiarrhythmics: 1.6%  Antidepressants: 16.6%  Anti-Parkinson: 0.4%  Antipsychotics: 1.6%  Anxiolytics: 6.4%  Hypnotics: 4.4%  Antihypertensives: 64.3% | Not reported | Not reported |
| Giorda et al. 2015 | Italian Region of Piedmont | Any admission for HF:  Cases: 52.6%  Controls: 52.6%  Incident HF:  Cases: 49.6%  Controls: 49.6%  Re-admission for HF:  Cases: 52.9%  Controls: 52.9%  All-cause mortality:  Cases: 50.2%  Controls: 50.2% | Any admission for HF:  Cases: 78.0 (8.3)^a^  Controls: 77.9 (8.4)^a^  Incident HF:  Cases: 78.3 (8.4)^a^  Controls: 78.2 (8.4)^a^  Re-admission for HF:  Cases: 77.9 (8.4) ^a^  Controls: 77.9 (8.5)^a^  All-cause mortality  Cases: 80.2 (9.1)^a^  Controls: 79.9 (9.0)^a^ | Ischaemic heart disease  Any admission for HF:  Cases: 23.1%  Controls: 7.0%  Incident HF:  Cases: 12.2%  Controls: 7.6%  Re-admission for HF  Cases: 16.3%  Controls: 13.7%  All-cause mortality  Cases: 11.2%  Controls: 6.3% | Any admission for HF:  Cases:  Glimepiride or glibenclamide: 10.5%  Insulin: 36.7%  Controls:  Glimepiride or glibenclamide: 11.5%  Insulin: 16.5%  Incident HF:  Cases:  Glimepiride or glibenclamide: 12.7%  Insulin: 30.2%  Controls:  Glimepiride or glibenclamide: 11.3%  Insulin: 16.7%  Re-admission for HF  Cases:  Glimepiride or glibenclamide: 10.0%  Insulin: 42.3%  Controls:  Glimepiride or glibenclamide: 9.8%  Insulin: 25.9%  All-cause mortality  Cases:  Glimepiride or glibenclamide: 9.0%  Insulin: 37.0%  Controls:  Glimepiride or glibenclamide: 12.1%  Insulin: 16.9% | Not reported | Not reported |
| Mistry et al. 2011 | Caucasian: 59.7%  Asian: 30.6%  Black: 9.7 | 52.8% | 73.0 (5.9) ^a^ | not reported | Not reported | Not reported | Not reported |
| Ou et al. 2015 | Taiwan | P using DPP-4 inhibitor: 53.9%  P using SU: 54.0% | P using DPP-4 inhibitor: 57.9 (12.3) ^a^  P using SU: 57.8 (12.3) ^a^ | P using DPP-4 inhibitor:  Coronary artery disease: 36.1%  Cerebrovascular disease: 20.9%  MI: 4.0%  Hypertension: 72.2%  HF: 8.6%  Peripheral vascular disease: 4.8%  Peptic ulcer disease: 26.6%  CKD: 11.3%  Liver disease: 39.8%  AF: 2.8%  Dyslipidemia: 74.7%  Valvular heart disease: 9.4%  Cancer: 11.9%  Autoimmune disease: 4.7%  P using SU: Coronary artery disease: 35.8%  Cerebrovascular disease: 21.1%  MI: 4.2%  Hypertension: 72.0%  HF: 8.7%  Peripheral vascular disease: 5.0%  Peptic ulcer disease: 26.4%  CKD: 11.2%  Liver disease: 39.8%  AF: 2.9%  Dyslipidemia: 74.8%  Valvular heart disease: 9.3%  Cancer: 11.9%  Autoimmune disease: 4.6% | P using DPP-4 inhibitor:  α-blocker: 1.7%  ACEI or ARB: 21.9%  Beta-blocker: 11.2%  CCB: 19.7%  Diuretic: 6.7%  Other antihypertensive drug: 0.6%  Antiplatelet agent: 13.4%  Warfarin: 0.6%  Steroid: 4.6%  Nitrate: 2.5%  NSAID: 14.9%  PPI: 2.1%  Statin: 12.3%  SSRI: 1.0%  P using SU:  α-Blocker: 1.7%  ACEI or ARB: 22.2%  Beta-blocker: 11.1%  CCB: 19.9%  Diuretic: 6.9%  Other antihypertensive drug: 0.6%  Antiplatelet agent: 13.4%  Warfarin: 0.7%  Steroid: 4.6%  Nitrate: 2.5%  NSAID: 14.9%  PPI: 2.0%  Statin: 12.3%  SSRI: 1.0% | No reported | Not reported |
| Penfornis et al. 2012 | Primary care  France | P using DPP-4 inhibitors: 62.1%  P using COAD: 56.4% | P using DPP-4 inhibitors: 71.0 (5.0) ^a^  P using COAD: 71.1 (5.2) ^a^ | P using DPP-4 inhibitors:  Microvascular complication: 27.0%  Macrovascular complication: 29.3%  Hypertension: 83.4%  Dyslipidaemia: 77.6%  P using COAD:  Microvascular complication: 31.5%  Macrovascular complication: 23.7%  Hypertension: 83.7%  Dyslipidaemia: 72.8% | P using DPP-4 inhibitors:  At least 1 antihypertensive: 82.9%  At least 1 SRA inhibitor: 70.1%  At least 1 hypolipidemic agent: 72.1%  Statin/Fibrates: 66.1%/6.6%  Platelet inhibitors: 37.2%  P using COAD:  At least 1 antihypertensive: 83.2%  At least 1 SRA inhibitor: 72.0%  At least 1 hypolipidemic agent: 71.7%  Statin/Fibrates: 61.9%/8.6%  Platelet inhibitors: 36.6% | Not reported | Not reported |
| Shih et al. 2015 | Taiwan | Cases: 53.7%  Controls: 53.7% | Cases: 78.3 (7.2)^a^  Controls: 78.4 (7.1)^a^ | Cases:  Hypertension: 85.8%  Coronary artery disease: 55.2%  MI: 8.5%  HF: 29.9%  Dyslipidemia: 63.3%  Cerebrovascular disease: 50.7%  Chronic liver disease: 44.2%  CKD: 32.3%  Autoimmune disease: 5.8%  Controls:  Hypertension: 85.3%  Coronary artery disease: 61.1%  MI: 8.7%  HF: 27.8%  Dyslipidemia: 73.2%  Cerebrovascular disease: 46.7%  Chronic liver disease: 49.0%  CKD: 35.1%  Autoimmune disease: 7.2% | Not reported | Not reported | Not reported |
| Sicras-Mainar and Navarro-Artieda 2014 | Spain | P using metformin + vildagliptin: 49.1%  P using metformin + SU: 50.5% | P using metformin + vildagliptin: 73.6 (6.1) ^a^  P using metformin + SU: 74.4 (6.2) ^a^ | P using metformin + vildagliptin:  Hypertension: 73.7%  Dyslipidaemia: 63.3%  Cardiovascular event: 28.1%  Obesity: 23.3%  Cerebrovascular accident: 18.9%  P using metformin + SU  Hypertension: 76.3%  Dyslipidaemia: 61.2%  Cardiovascular event: 31.2%  Obesity: 23.2%  Cerebrovascular accident: 19.8% | Not reported | Not reported | Not reported |
| Tziomalos et al. 2015 | Hospital department of internal medicine in Greece | P using DPP-4 inhibitors: 44.4%  P using other antidiabetic  agents: 37.0% | P using DPP-4 inhibitors: 76.0 (7.6)^a^  P using other antidiabetic agents: 78.6 (6.1)^a^ | P using DPP-4 inhibitors:  Hypertension: 100%  AF: 37.0%  Coronary heart disease 40.7%  Previous ischaemic stroke: 59.3%  CHF: 25.9%  P using other antidiabetic agents:  Hypertension: 84.9%  AF: 46.6%  Coronary heart disease: 34.2%  Previous ischaemic stroke: 43.8%  CHF: 32.9% | Not reported | Modified Rankin Scale score at hospital discharge | Not reported |
| Viljoen et al. 2013 | Primary and secondary care  UK | P using DPP-4 inhibitor: 64%  P never treated with DPP-4 inhibitors: 54% | P using DPP-4 inhibitor: 70 (60-92) ^b^  P never treated with DPP-4 inhibitors: 77 (60- 95) ^b^ | P using DPP-4 inhibitor:  Retinopathy: 28%  Neuropathy: 14%  Ischemic heart disease: 21%  Stroke: 8%  Peripheral artery disease: 4%  P never treated with DPP-4 inhibitors:  Retinopathy: 20%  Neuropathy: 31%  Ischemic heart disease: 29%  Stroke: 10%  Peripheral artery disease: 8% | Not reported | Not reported | Not reported |
| Yu et al. 2015 | Primary care  Hospital  UK | Cases: 58.1%  Controls: 52.5% | Cases: 73.3 (11.0)^a^  Controls: 73.2 (10.9)^a^ | Cases:  Neuropathy: 18.0%  Renal disease: 34.4%  Retinopathy: 29.2%  AF: 21.3%  Cancer: 19.5%  COPD: 28.1%  Coronary arterial disease: 48.5%  Coronary revascularisation: 9.7%  Dyslipidemia: 36.9%  Hypertension: 76.1%  MI: 18.2%  Peripheral arteriopathy: 17.6%  Peripheral vascular disease: 15.3%  Stroke: 10.0%  Controls:  Neuropathy: 17.5%  Renal disease: 26.1%  Retinopathy: 28.4%  AF: 9.6%  Cancer: 16.1%  COPD: 14.1%  Coronary arterial disease: 31.6%  Coronary revascularisation: 6.3%  Dyslipidemia: 32.5%  Hypertension: 70.0%  MI: 8.6%  Peripheral arteriopathy: 9.0%  Peripheral vascular disease: 7.9%  Stroke: 7.3% | Cases:  ACE-I: 51.3%  ARB: 16.9%  Beta-blockers: 36.0%  CCB: 44.6%  Diuretics: 58.7%  Fibrates: 2.2%  Statins: 68.8%  Aspirin: 52.8%  Other NSAID: 17.9%  Controls:  ACE-I: 44.9%  ARB: 16.6%  Beta-blockers: 28.6%  CCB: 35.2%  Diuretics: 42.0%  Fibrates: 1.9%  Statins: 68.2%  Aspirin: 46.2%  Other NSAID: 18.0% | Not reported | Not reported |

ACE-I: angiotensin-converting-enzyme inhibitor, AF: atrial fibrillation, ARB: angiotensin receptor blocker, ASA: acetyl-salicylic acid, CCB: calcium channel blockers, CHF: congestive heart failure, CKD: chronic kidney disease, COAD: conventional oral antidiabetic drugs, COPD: chronic obstructive pulmonary disease, CRD: chronic renal disease, CV: cardiovascular, HF: heart failure, MI: myocardial infarction, NA: not applicable, NAM: no anti-hyperglycaemic medications, NSAID: non-steroidal anti-inflammatory drugs, OAD: oral antidiabetic agents, OAM: other anti-hyperglycaemic medications, P: participants, PPI: proton pump inhibitors, SRA inhibitor : not defined by Penfornis Et al. (2012), SSRI: selective serotonin reuptake inhibitor, SU: sulfonylureas, TZD: thiazolidinediones, UK: United Kingdom, USA: United States of America.

^a^ mean age in years (standard deviation), ^b^ median age in years (range), ^c^ median age in years (interquartile range), ^d^ see Driessen et al. (2014) for a full list of comorbidites.
