# Supplementary material for: Effectiveness and safety of dipeptidyl peptidase 4 inhibitors in the management of type 2 diabetes in older adults: a systematic review and development of recommendations to reduce inappropriate prescribing
Source: BMC Geriatr. 2017 Oct 16;17(Suppl 1):226. doi: 10.1186/s12877-017-0571-8 (PMC5647559; doi:10.1186/s12877-017-0571-8)
Supplement: Supplementary file 4 — Additional evidence for recommendation. Additional evidence for recommendation. (DOCX 15 kb) [file 12877_2017_571_MOESM4_ESM.docx]

**Table S2 - Additional evidence for recommendation**

| **Source** | **Type of evidence** | **Relevant outcomes** |
| --- | --- | --- |
| Weir DL, McAlister FA, Senthilselvan A, Minhas-Sandhu JK, Eurich DT. Sitagliptin use in patients with diabetes and heart failure: a population-based retrospective cohort study. JACC Heart failure. 2014;2(6):573-82. doi:10.1016/j.jchf.2014.04.005. | Observational study | Increased risk of hospitalisations for heart failure in people under sitagliptin compared to people under other anti-diabetic treatments (OR 1.84, 95% CI 1.16 to 2.92) |
| Wu S, Hopper I, Skiba M, Krum H. Dipeptidyl peptidase-4 inhibitors and cardiovascular outcomes: meta-analysis of randomised clinical trials with 55,141 participants. Cardiovascular therapeutics. 2014;32(4):147-58. doi:10.1111/1755-5922.12075. | Meta-analysis | Increased risk of heart failure outcomes in people under DPP-4 inhibitors compared to people under other anti-diabetic treatments or placebo (RR 1.16, 95% CI 1.01 to 1.33) |
| Egan AG, Blind E, Dunder K, de Graeff PA, Hummer BT, Bourcier T et al. Pancreatic safety of incretin-based drugs--FDA and EMA assessment. The New England journal of medicine. 2014;370(9):794-7. doi:10.1056/NEJMp1314078. | FDA and EMA assessment | Uncertainties of long term pancreatic safety with DPP-4 inhibitors |
| Schweizer A, Dejager S, Foley JE, Couturier A, Ligueros-Saylan M, Kothny W. Assessing the cardio-cerebrovascular safety of vildagliptin: meta-analysis of adjudicated events from a large Phase III type 2 diabetes population. Diabetes, obesity & metabolism. 2010;12(6):485-94. doi:10.1111/j.1463-1326.2010.01215.x. | Pooled analysis | No statistically significant differences between vildagliptin compared to other anti-diabetic treatments or placebo for a composite endpoint which included acute coronary syndrome, transient ischaemic attack, stroke, myocardial infarction, cardiovascular and cerebrovascular death in people ≥65 years old (RR 1.04, 95% CI 0.62 to 1.73) |

OR: odds ratio, CI: confidence interval, DPP-4: dipeptidyl peptidase-4, RR: risk ratio, FDA: Food and Drug Administration, EMA: European Medicines Agency.
